# Clinical Outcomes of Combined TAVR-PCI Versus SAVR-CABG in Patients with Aortic Stenosis and Coronary Artery Disease: A Systematic Review and Meta-Analysis

**DOI:** 10.3390/jcm15197556

**Published:** 2026-09-29

**Authors:** Ricardo Antonio Rodriguez Mejia, Mathhar Aldaoud, Ifeanyi Momodu, Merina Dongol, Girum Feyissa, Kelechi Aruogu, Zamaraq M. Bhatti, Abdul Haseeb Riaz, Nafisa Saleem

**Affiliations:** 1 Cape Fear Valley Medical Center, Methodist University Cape Fear Valley Health School of Medicine, Fayetteville, NC 28304, USA; 2 Department of Cardiology, Faculty of Medicine, University of Jordan, Amman 11942, Jordan

**Keywords:** aortic stenosis, coronary artery disease, transcatheter aortic valve replacement, percutaneous coronary intervention, surgical aortic valve replacement, coronary artery bypass grafting, systematic review, meta-analysis

## Abstract

**Background/Objectives:** Coronary artery disease coexists with severe aortic stenosis in about half of patients. Surgical aortic valve replacement with coronary artery bypass grafting (SAVR+CABG) is guideline-recommended, but transcatheter aortic valve replacement with percutaneous coronary intervention (TAVR+PCI) is increasingly used. Prior meta-analyses double-counted overlapping populations and did not report randomized evidence separately; we re-examined the comparison after correcting both. **Methods:** PubMed, Embase, the Cochrane Central Register of Controlled Trials (CENTRAL), Web of Science and ClinicalTrials.gov were searched up to July 2026 for randomized and comparative observational studies, excluding reports with overlapping populations. Outcomes were pooled with random-effects models, stratified by study design, and re-analysed without administrative databases. Certainty was rated with the Grading of Recommendations Assessment, Development and Evaluation (GRADE) approach. **Results:** Fourteen studies were included (two randomized trials, nine cohorts, three administrative databases), comprising 154,892 patients (28,569 TAVR+PCI; 126,323 SAVR+CABG). TAVR+PCI was associated with lower early mortality (odds ratio (OR) 0.60, 95% confidence interval (CI) 0.44–0.84) and less acute kidney injury (OR 0.34, 0.20–0.57), but greater pacemaker implantation (OR 2.18, 1.93–2.46) and major vascular complications (OR 3.38, 1.65–6.89). Stroke, myocardial infarction and one-year mortality (OR 0.80, 0.58–1.10) did not differ; bleeding results were inconsistent (I^2^ = 95%). Over 12–72 months, mortality was higher with TAVR+PCI (hazard ratio (HR) 1.38, 1.28–1.49; nine observational studies), unchanged without the largest cohort. Administrative databases drove heterogeneity but not the direction of any early outcome. Certainty was moderate for pacemaker implantation and low or very low otherwise; randomized data were too limited to confirm these findings. **Conclusions:** In predominantly observational evidence, TAVR+PCI was associated with lower early risk but higher mortality over longer follow-up. This trade-off supports individualized Heart Team decisions rather than the superiority of either strategy. Adequately powered randomized trials with long-term follow-up are needed.

## 1. Introduction

Coronary artery disease (CAD) coexists with severe aortic stenosis in about half of patients referred for valve intervention [1,2]. Guidelines recommend surgical aortic valve replacement with concomitant coronary artery bypass grafting (SAVR+CABG) when both require treatment [3,4], but this rests largely on surgical series from before the transcatheter era. As transcatheter aortic valve replacement (TAVR) has expanded across the risk spectrum, a fully percutaneous strategy of TAVR with percutaneous coronary intervention (TAVR+PCI) has become a routine alternative, even though patients with significant CAD were largely excluded from the pivotal TAVR trials.

Randomised evidence is limited to a prespecified SURTAVI subgroup of 332 intermediate-risk patients with non-complex CAD, which showed similar 2-year outcomes with either strategy [5], and the TCW trial, the first dedicated comparison, which found non-inferiority of fractional flow reserve (FFR)-guided TAVR+PCI at 1 year but was stopped after 172 patients [6]. The remaining evidence is observational, ranging from single-centre propensity-matched cohorts to large administrative databases, and suggests lower early mortality, bleeding, and kidney injury with TAVR+PCI, offset by greater pacemaker implantation and vascular complications, and possibly higher mortality beyond the first year [7,8,9].

Several meta-analyses have pooled these data, including three published in 2026 [10,11,12,13,14]. They reach similar conclusions but share limitations. Each includes studies drawn from overlapping populations, two analyses of the same national inpatient sample over the same years [15,16], a single-centre cohort nested within a later multicentre registry [7,17], and a national administrative dataset encompassing the centres of a concurrent clinical registry [9,18] so the same patients are counted more than once. Randomised and observational data have been combined without a separately reported randomised stratum, outcomes have been pooled across differing definitions and horizons, and the safety outcomes that drove the TCW result, bleeding and vascular complications, have not been synthesised with attention to how they were defined.

We therefore performed a systematic review and meta-analysis using a deduplicated set of comparative studies, stratified by design with a separately reported randomised stratum, with uniform outcome horizons and definition-based subgroups, and with mortality assessed early, at 1 year, and over the longest follow-up. Our aim was to determine whether the conclusions of prior syntheses hold after these corrections.

## 2. Methods

### 2.1. Protocol and Reporting

This systematic review and meta-analysis was conducted according to the Preferred Reporting Items for Systematic Reviews and Meta-Analyses (PRISMA) 2020 statement [19] and the Cochrane Handbook [20]. The PRISMA 2020 checklist is provided as Supplementary File S1.

### 2.2. Eligibility Criteria

Studies were eligible if they (1) enrolled adults with severe aortic stenosis and concomitant coronary artery disease requiring revascularization; (2) compared TAVR with PCI (staged or concomitant) against SAVR with concomitant CABG as complete treatment strategies; (3) reported at least one outcome of interest with per-arm event counts or an adjusted effect estimate; and (4) were randomised controlled trials or comparative observational studies published in full in a peer-reviewed journal. Hybrid strategies (TAVR with surgical revascularization or SAVR with PCI), single-arm series, case reports, conference abstracts, reviews, and meta-analyses were excluded. No language or date restrictions were applied.

**Figure 1:**
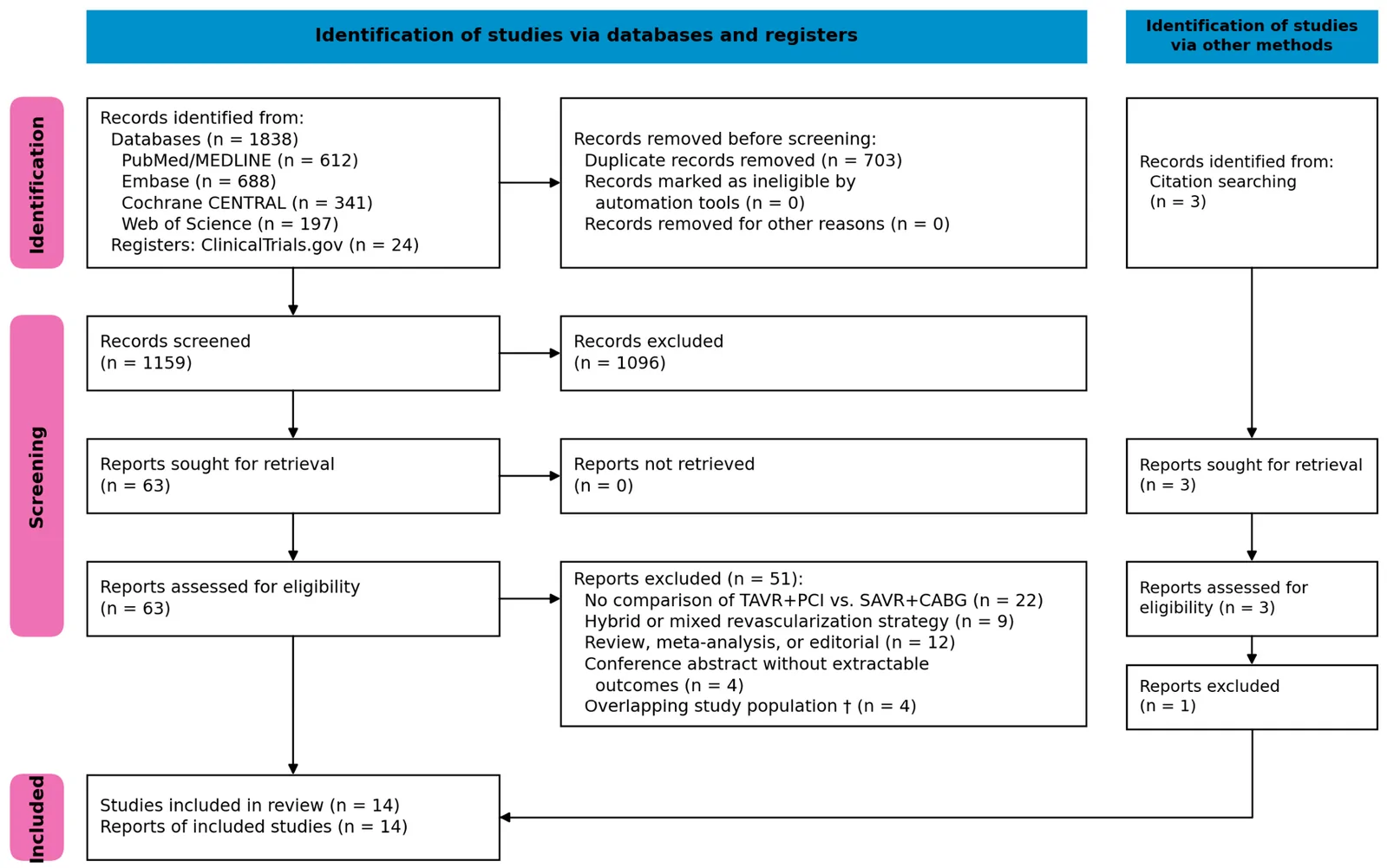
PRISMA Flow chart. † Overlapping populations: Abugroun 2020 [15] (NIS 2016–2017, within Patlolla 2022 [16]); Potratz 2025 [17] (Bad Oeynhausen 2016–2019, within Stundl 2026 [7]); Taghiyev 2025 (Thorac Cardiovasc Surg) [21] and Taghiyev 2026 (BMC Cardiovasc Disord) [22], same cohort as Taghiyev 2025 (J Card Surg) [23]; McInerney 2023 [18] (Spanish national discharge data 2016–2019, overlapping Amat-Santos 2024 [9]). NIS, National Inpatient Sample.

Where two or more reports drew on overlapping patient populations, the same database over overlapping years, the same institution over overlapping enrolment periods, or a national dataset encompassing the centres of a concurrent registry, only one was retained, selected by larger sample size, longer enrolment, and completeness of outcome reporting, in that order. Reports excluded for this reason are listed in the PRISMA diagram (Figure 1).

### 2.3. Search Strategy

PubMed/MEDLINE, Embase, the Cochrane Central Register of Controlled Trials, Web of Science, and ClinicalTrials.gov were searched from inception to July 2026. The PubMed strategy combined terms for the population and interventions: (“aortic stenosis” OR “aortic valve stenosis”) AND (“coronary artery disease” OR “coronary revascularization” OR “myocardial revascularization”) AND (“transcatheter aortic valve” OR TAVR OR TAVI) AND (“percutaneous coronary intervention” OR PCI) AND (“surgical aortic valve replacement” OR SAVR) AND (“coronary artery bypass” OR CABG). The full strategy for each database is presented in Supplementary Table S1. Reference lists of included studies and of three recent meta-analyses [10,11,12] were hand-searched.

### 2.4. Selection Process

Records were de-duplicated in Zotero (Corporation for Digital Scholarship, Vienna, VA, USA). Two reviewers independently screened titles and abstracts, then full texts, against the eligibility criteria. Disagreements were resolved by discussion, with a third reviewer adjudicating if needed. The selection process is summarized in a PRISMA 2020 flow diagram (Figure 1).

### 2.5. Data Extraction

Two reviewers independently extracted data into a prespecified form and reconciled discrepancies against the source tables. For each study, we recorded design, data source, enrolment period, adjustment method, cohort analysed, sample size per arm, baseline age, sex, surgical risk score, CAD complexity, PCI timing, follow-up duration, and outcome definitions. Where a study reported both matched (or weighted) and unmatched cohorts, the matched cohort was extracted. For administrative databases reporting only percentages, event counts were back-calculated from the published proportions and denominators.

Outcomes were extracted as per-arm event counts at prespecified horizons. Early outcomes (all-cause mortality, stroke, myocardial infarction, acute kidney injury, permanent pacemaker implantation, major or life-threatening bleeding, major vascular complications) were extracted at the in-hospital or 30-day horizon; where a study reported only a later horizon, the outcome was not extracted from that study. One-year all-cause mortality was extracted as observed deaths at 12 months. Repeat coronary revascularization was extracted at longest reported follow-up. For studies using Valve Academic Research Consortium-2 (VARC-2) definitions [24], life-threatening and major bleeding were summed to align with the VARC-3 major bleeding category [25]. Reported hazard ratios for all-cause mortality over follow-up were extracted with their 95% confidence intervals and standardised so that SAVR+CABG was the reference. Where no hazard ratio was reported but a Kaplan–Meier curve with numbers at risk was published, the hazard ratio was estimated from the curve using the methods of Tierney et al. [26]. Covariate-adjusted odds ratios for early outcomes were extracted where reported. Outcome definitions and the horizon used for each study are tabulated in Supplementary Tables S2 (Panels A and B) and S3.

### 2.6. Risk of Bias

Randomised trials were assessed with the Cochrane risk-of-bias tool for randomised trials (RoB 2) [27] and observational studies with the Newcastle–Ottawa Scale [28], independently by two reviewers. Study-level judgements are reported in Supplementary Tables S4 and S5.

### 2.7. Methods for Synthesis of Data

The synthesis comprised 14 studies and 154,892 patients (28,569 TAVR+PCI; 126,323 SAVR+CABG), counted as the per-arm size of the cohort analysed in each study and including weighted national estimates from administrative databases. Binary outcomes were pooled as odds ratios (ORs) with 95% confidence intervals (CIs) using a random-effects model with restricted maximum-likelihood (REML) estimation of between-study variance. Zero-event cells were handled with a 0.5 continuity correction; the randomised stratum was additionally analysed with the Peto method. Hazard ratios (HRs) were pooled on the log scale using the inverse-variance random-effects method. Heterogeneity was quantified with Cochran’s Q and I^2^ [29], and τ^2^ is reported for every analysis.

Every analysis was stratified by study design (randomised trial; adjusted cohort propensity-matched, inverse-probability-weighted, or regression-adjusted; unadjusted cohort; administrative database), with the randomised stratum reported separately and a test for subgroup difference. Prespecified additional subgroups were early mortality definition (in-hospital vs. 30-day); outcome definition (VARC-based vs. administrative-coded vs. other) for acute kidney injury, bleeding, and vascular complications; inclusion of transient ischaemic attack for stroke; and reported vs. estimated hazard ratio. Sensitivity analyses excluded (1) administrative databases, (2) hazard ratios estimated from survival curves, (3) hazard ratios derived from unadjusted curves, and (4) each study in turn (leave-one-out); a further analysis pooled the most adjusted available estimate for early outcomes, combining covariate-adjusted odds ratios where reported with crude odds ratios otherwise. Small-study effects were assessed by using a funnel plot and Egger’s test [30] for outcomes with at least ten studies. Meta-regression was not performed given the number of studies per outcome. Repeat revascularization was analysed as exploratory only.

Certainty of evidence was rated with the Grading of Recommendations Assessment, Development and Evaluation (GRADE) approach [31] separately for the randomised and observational strata of each outcome, and the stratum on which each conclusion rests is stated in the summary-of-findings table (Supplementary Table S6). Analyses were performed in IBM SPSS Statistics version 30 (IBM Corp., Armonk, NY, USA).

## 3. Results

### 3.1. Study Selection

Fourteen studies were included (Figure 1): two randomized trials [5,6], nine observational cohorts [7,9,23,32,33,34,35,36,37], and three administrative databases [8,16,38]. Together they comprised 154,892 patients, 28,569 treated with TAVR+PCI and 126,323 with SAVR+CABG: 500 in randomized trials (260 and 240), 3014 in observational cohorts (1298 and 1716), and 151,378 in administrative databases (27,011 and 124,367, including weighted national estimates). Per-study sample sizes are given in Supplementary Table S2.

### 3.2. Early All-Cause Mortality

**Figure 2:**
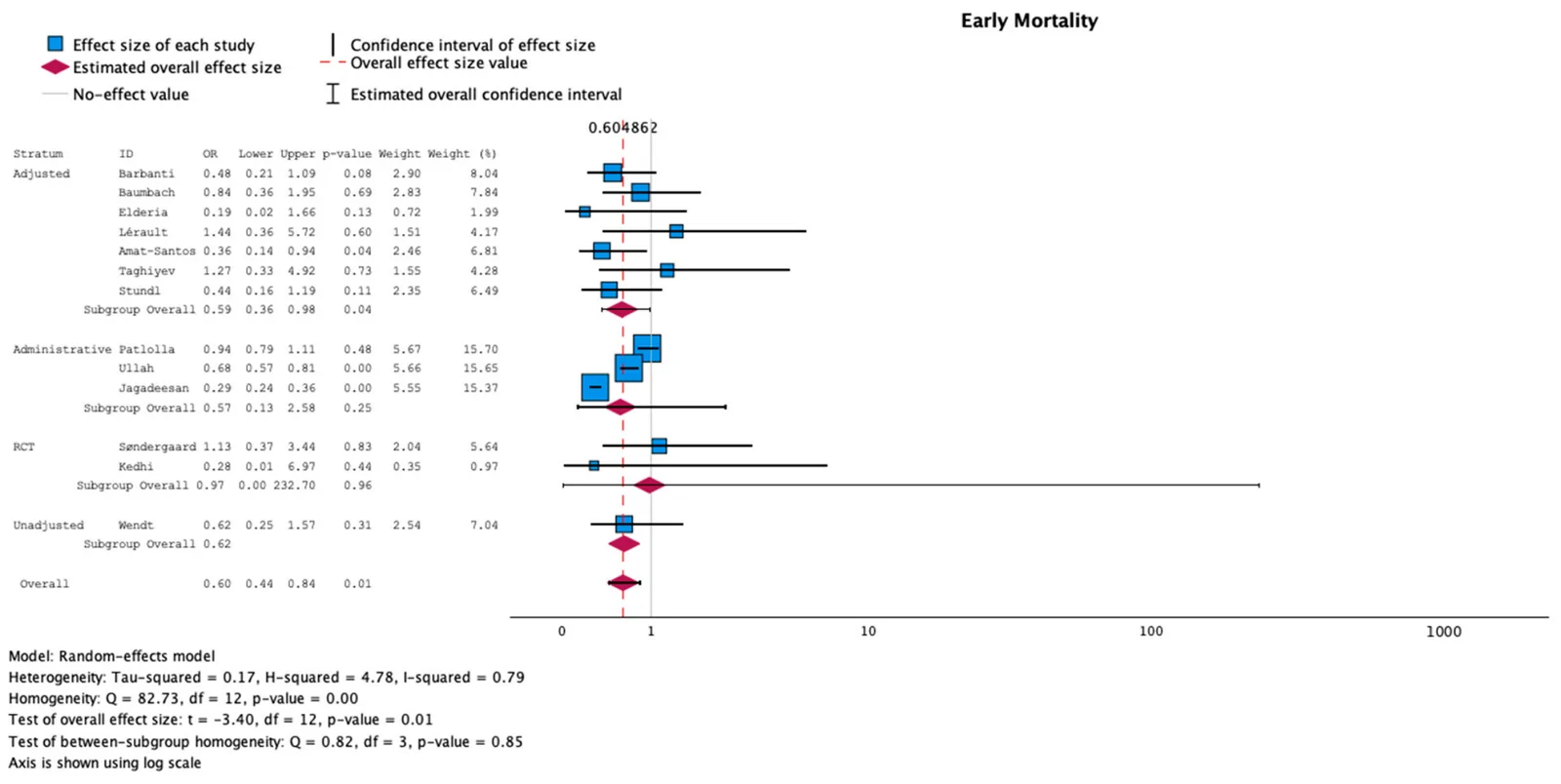
Early (in-hospital or 30-day) all-cause mortality after TAVR+PCI versus SAVR+CABG, stratified by study design. Random-effects model (REML); SAVR+CABG is the reference, and OR < 1 favours TAVR+PCI. Diamonds show pooled estimates for each stratum and overall. TAVR+PCI, transcatheter aortic valve replacement with percutaneous coronary intervention; SAVR+CABG, surgical aortic valve replacement with coronary artery bypass grafting; OR, odds ratio; CI, confidence interval; REML, restricted maximum likelihood.

Thirteen studies (28,413 TAVR+PCI and 126,167 SAVR+CABG patients, including weighted national estimates) reported in-hospital or 30-day mortality. TAVR+PCI was associated with lower early mortality (OR 0.60, 95% CI 0.44–0.84; I^2^ = 79%; Figure 2). Heterogeneity arose almost entirely from the three administrative databases, whose estimates ranged from OR 0.94 (0.79–1.11; Patlolla et al. [16]) to 0.29 (0.24–0.36; Jagadeesan et al. [8]) and whose cohorts were identified by differing International Classification of Diseases (ICD) code algorithms. Excluding them left the estimate unchanged and eliminated heterogeneity (10 studies; OR 0.63, 95% CI 0.44–0.89; I^2^ = 0%; Figure S1). Egger’s test did not indicate small-study effects in either the full set (*p* = 0.98) or the non-administrative studies (*p* = 0.88). By design, the association was significant in adjusted cohorts (OR 0.59, 0.36–0.98), directionally similar in administrative databases (OR 0.57, 0.13–2.58) and the single unadjusted cohort (OR 0.62, 0.25–1.57), and absent in the two randomized trials (OR 0.97, 0.00–232.70), which contributed few events; the test for subgroup differences was not significant (*p* = 0.85) but had limited power. 

### 3.3. All-Cause Mortality over Follow-Up

**Figure 3:**
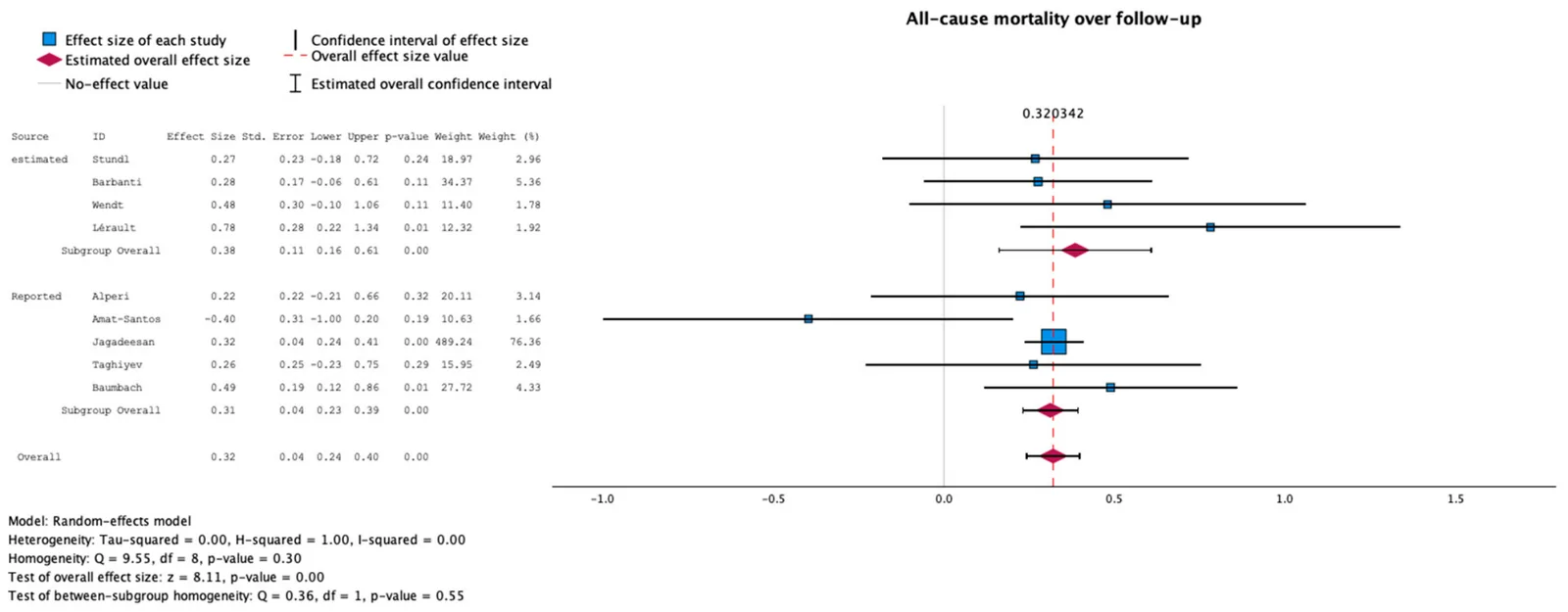
All-cause mortality over follow-up after TAVR+PCI versus SAVR+CABG, by source of the hazard ratio (reported by the original authors or estimated from Kaplan–Meier data). Random-effects model (REML) on the log hazard ratio scale; SAVR+CABG is the reference, and values > 0 (HR > 1) favour SAVR+CABG. Diamonds show pooled estimates for each source and overall. HR, hazard ratio.

Nine observational studies contributed a hazard ratio for all-cause mortality (follow-up 12–72 months); five were reported by the original authors and four were estimated from Kaplan–Meier data. No randomized trial reported this outcome. TAVR+PCI was associated with higher mortality than SAVR+CABG (HR 1.38, 95% CI 1.28–1.49; I^2^ = 0%; Figure 3). Because the Medicare cohort (Jagadeesan et al. [8]) carried 76% of the weight, the analysis was repeated without it, with an unchanged result (8 studies; HR 1.37, 95% CI 1.17–1.61; I^2^ = 0%; Figure S2). Reported (HR 1.37, 95% CI 1.26–1.48) and estimated hazard ratios (HR 1.47, 95% CI 1.18–1.84) did not differ (*p* = 0.55), although reported hazard ratios alone were not significant once the Medicare cohort was excluded (HR 1.22, 95% CI 0.88–1.69; 4 studies). Restricting to studies with at least 24 months of follow-up gave a similar result (HR 1.38, 95% CI 1.28–1.50). By design, the direction was consistent in the administrative database (HR 1.38, 95% CI 1.27–1.51), the adjusted cohorts (7 studies; HR 1.35, 95% CI 1.13–1.61) and the single unadjusted cohort (HR 1.62, 95% CI 0.90–2.89).

### 3.4. One-Year All-Cause Mortality

**Figure 4:**
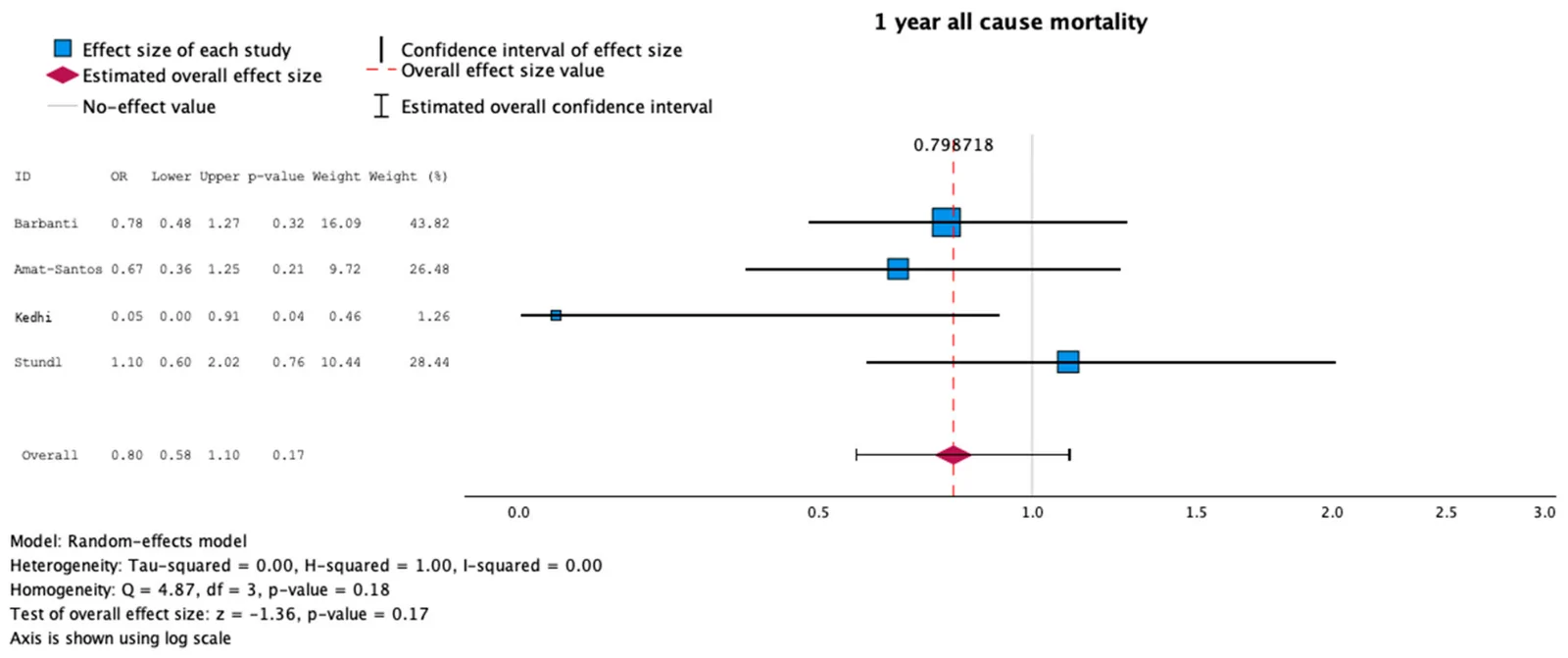
One-year all-cause mortality after TAVR+PCI versus SAVR+CABG. Random-effects model (REML); SAVR+CABG is the reference, and OR < 1 favours TAVR+PCI.

Four studies (735 TAVR+PCI and 721 SAVR+CABG patients) reported 1-year all-cause mortality. The difference between strategies was not significant (OR 0.80, 95% CI 0.58–1.10; *p* = 0.17), without heterogeneity (I^2^ = 0%; Figure 4). The only randomized trial [6], which was stopped early and recorded no deaths in the TAVR+PCI arm, favoured TAVR+PCI (OR 0.05, 95% CI 0.00–0.91) but carried 1.3% of the weight; the three adjusted cohorts ranged from OR 0.67 to 1.10, with none individually significant.

### 3.5. Pacemaker Implantation

**Figure 5:**
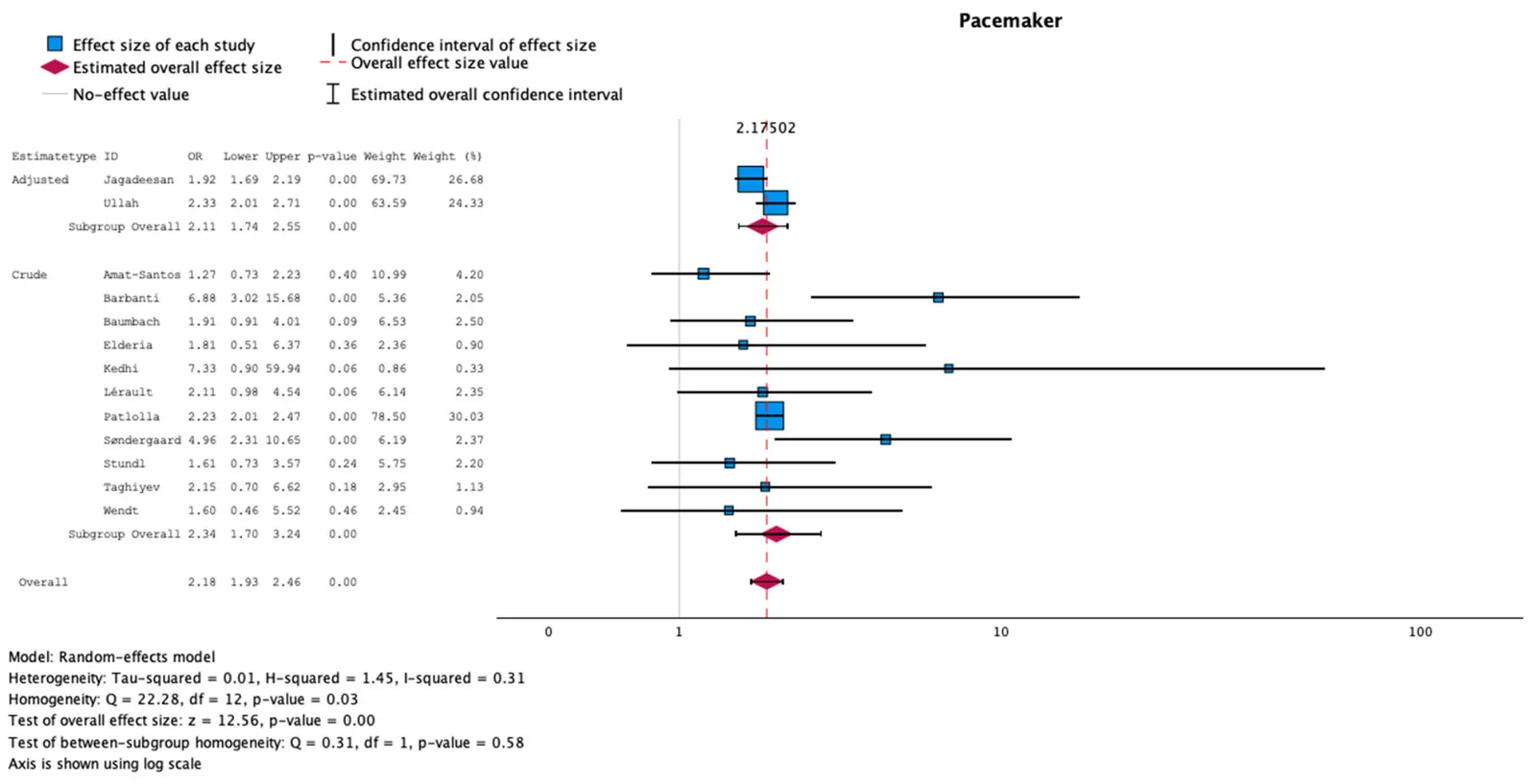
Permanent pacemaker implantation after TAVR+PCI versus SAVR+CABG, by type of estimate (covariate-adjusted or crude). Random-effects model (REML); SAVR+CABG is the reference, and OR > 1 favours SAVR+CABG. Diamonds show pooled estimates for each stratum and overall.

Thirteen studies reported new permanent pacemaker implantation. TAVR+PCI was associated with a higher risk than SAVR+CABG (OR 2.18, 95% CI 1.93–2.46; *p* < 0.001), with low-to-moderate heterogeneity (I^2^ = 31%; Figure 5). Every study had a point estimate above 1, ranging from OR 1.27 (Amat-Santos et al. [9]) to 7.33 (Kedhi et al. [6]). The association was similar for covariate-adjusted estimates (2 studies; OR 2.11, 95% CI 1.74–2.55) and crude estimates (11 studies; OR 2.34, 95% CI 1.70–3.24; *p* for subgroup difference = 0.58). Both randomized trials showed the same direction (Søndergaard et al. [5], OR 4.96, 95% CI 2.31–10.65; Kedhi et al. [6], OR 7.33, 95% CI 0.90–59.94).

### 3.6. Vascular Complications

**Figure 6:**
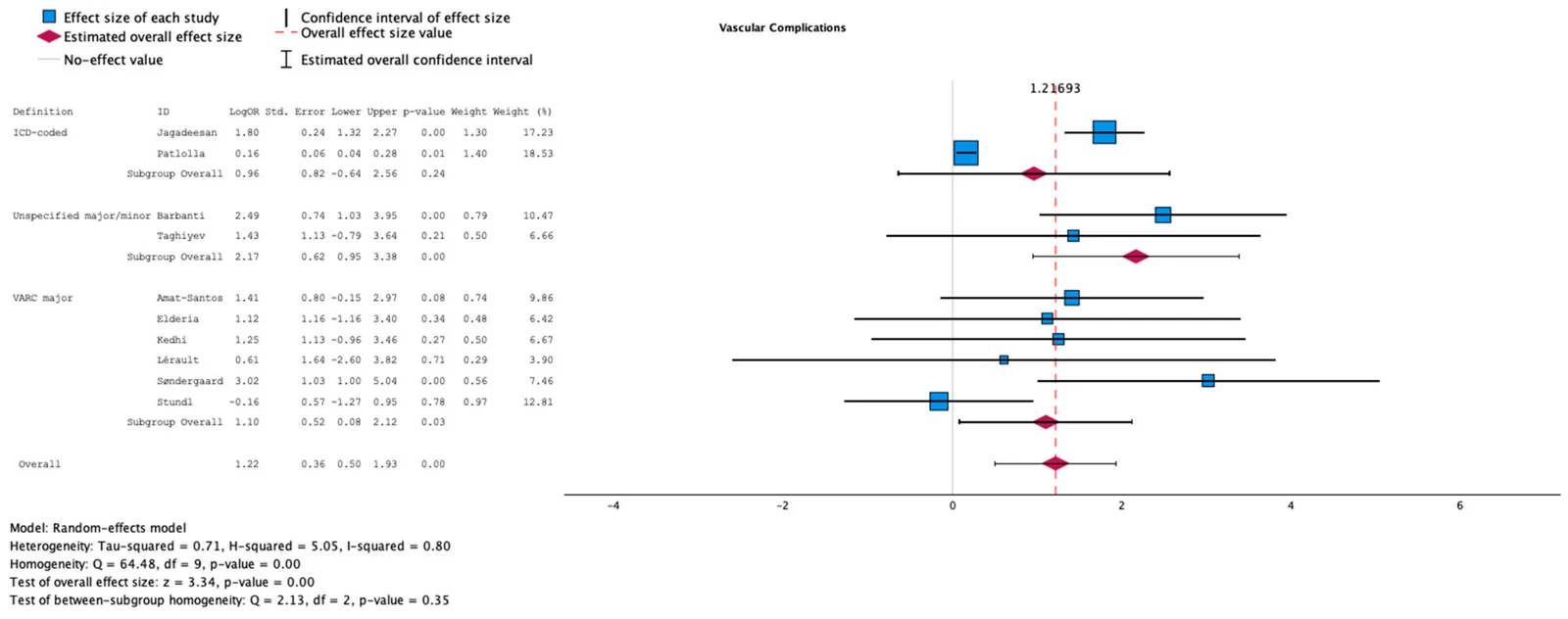
Major vascular complications after TAVR+PCI versus SAVR+CABG, by outcome definition. Random-effects model (REML) on the log odds ratio scale; SAVR+CABG is the reference, and values > 0 (OR > 1) favour SAVR+CABG.

Ten studies reported early vascular complications. TAVR+PCI was associated with a higher risk than SAVR+CABG (OR 3.38, 95% CI 1.65–6.89; *p* < 0.001), with substantial heterogeneity (I^2^ = 80%; Figure 6). Excluding the two administrative databases, whose ICD-coded estimates were discordant (Patlolla et al. [16], OR 1.17; Jagadeesan et al. [8], OR 6.02), gave a similar estimate with less heterogeneity (8 studies; OR 3.90, 95% CI 1.64–9.32; I^2^ = 43%; Figure S3). By outcome definition, the association was present in studies using VARC major vascular complications (6 studies; OR 3.00, 95% CI 1.08–8.33) and in those reporting unspecified major or minor complications (2 studies; OR 8.76, 95% CI 2.59–29.4), and directionally consistent in the administrative databases (OR 2.61, 95% CI 0.53–12.9; *p* for subgroup difference = 0.35).

### 3.7. Stroke

**Figure 7:**
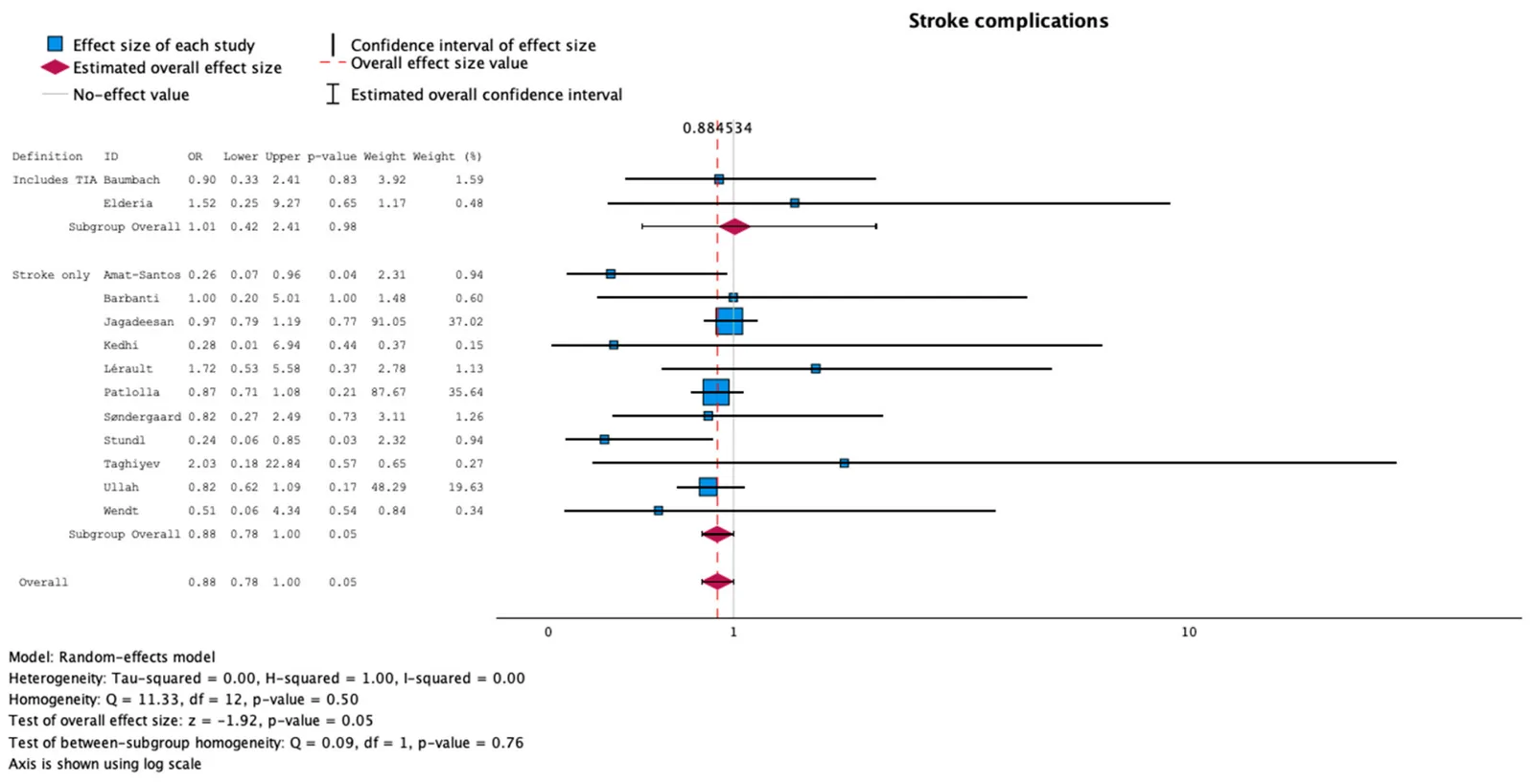
Early stroke after TAVR+PCI versus SAVR+CABG, by outcome definition. Random-effects model (REML); SAVR+CABG is the reference, and OR < 1 favours TAVR+PCI.

Thirteen studies reported early stroke. The difference between strategies was not significant (OR 0.88, 95% CI 0.78–1.00; *p* = 0.054), without heterogeneity (I^2^ = 0%; Figure 7). The three administrative databases carried 92% of the weight; excluding them gave a similar, less precise estimate (10 studies; OR 0.72, 95% CI 0.44–1.20; I^2^ = 16%; Figure S4). Results did not differ between studies reporting stroke alone (OR 0.88, 95% CI 0.78–1.00) and those including transient ischaemic attack (OR 1.01, 95% CI 0.42–2.41; *p* for subgroup difference = 0.76). Egger’s test did not indicate small-study effects (*p* = 0.38).

### 3.8. Major Bleeding

**Figure 8:**
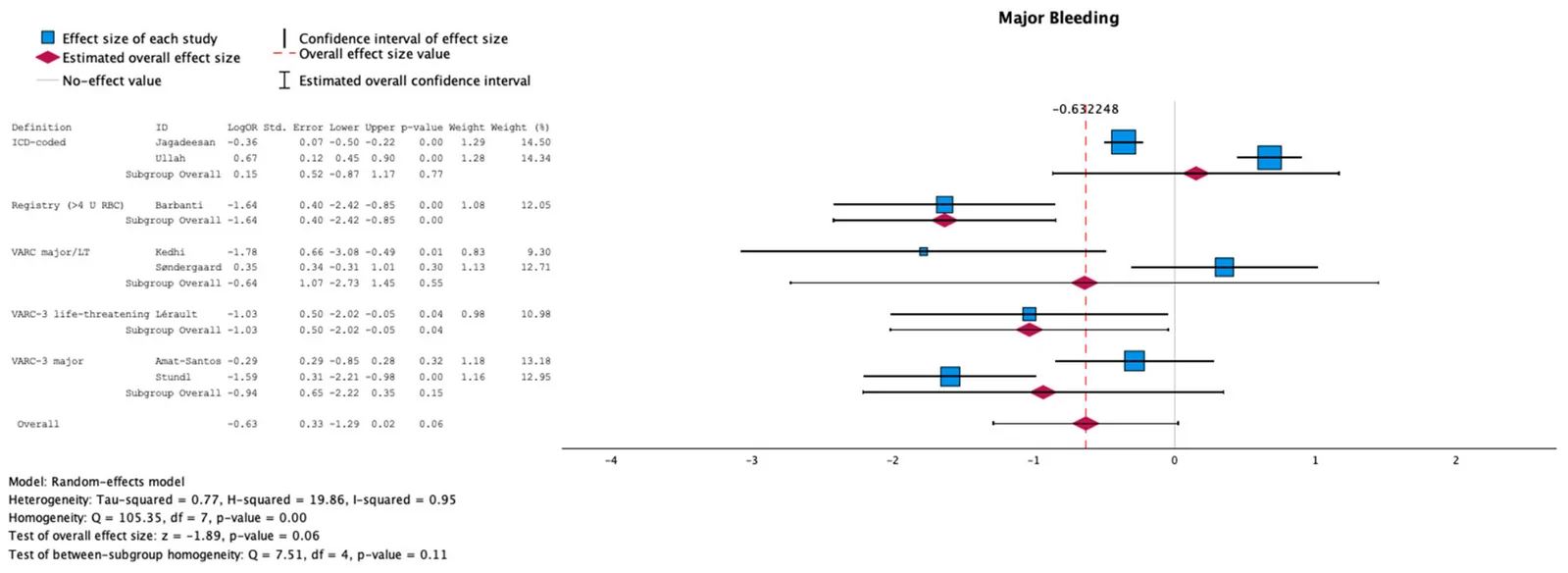
Early major or life-threatening bleeding after TAVR+PCI versus SAVR+CABG, by outcome definition. Random-effects model (REML) on the log odds ratio scale; SAVR+CABG is the reference, and values < 0 (OR < 1) favour TAVR+PCI.

Eight studies reported early major bleeding. The pooled difference was not significant (OR 0.53, 95% CI 0.28–1.02; *p* = 0.06), and heterogeneity was extreme (I^2^ = 95%; Figure 8). Estimates ranged from a marked reduction with TAVR+PCI (Kedhi et al. [6], OR 0.17; Barbanti et al. [33], OR 0.19; Stundl et al. [7], OR 0.20) to an increase (Ullah et al. [38], OR 1.95; Søndergaard et al. [5], OR 1.42), and the two administrative databases pointed in opposite directions. Excluding them, TAVR+PCI was associated with less bleeding, although heterogeneity remained substantial (6 studies; OR 0.39, 95% CI 0.19–0.79; I^2^ = 81%; Figure S5). Outcome definition did not explain the inconsistency (*p* for subgroup difference = 0.11 in the full set and 0.65 without administrative databases), and studies sharing the same definition gave discordant results. Small-study effects were not assessed because fewer than ten studies were available.

### 3.9. Acute Kidney Injury

**Figure 9:**
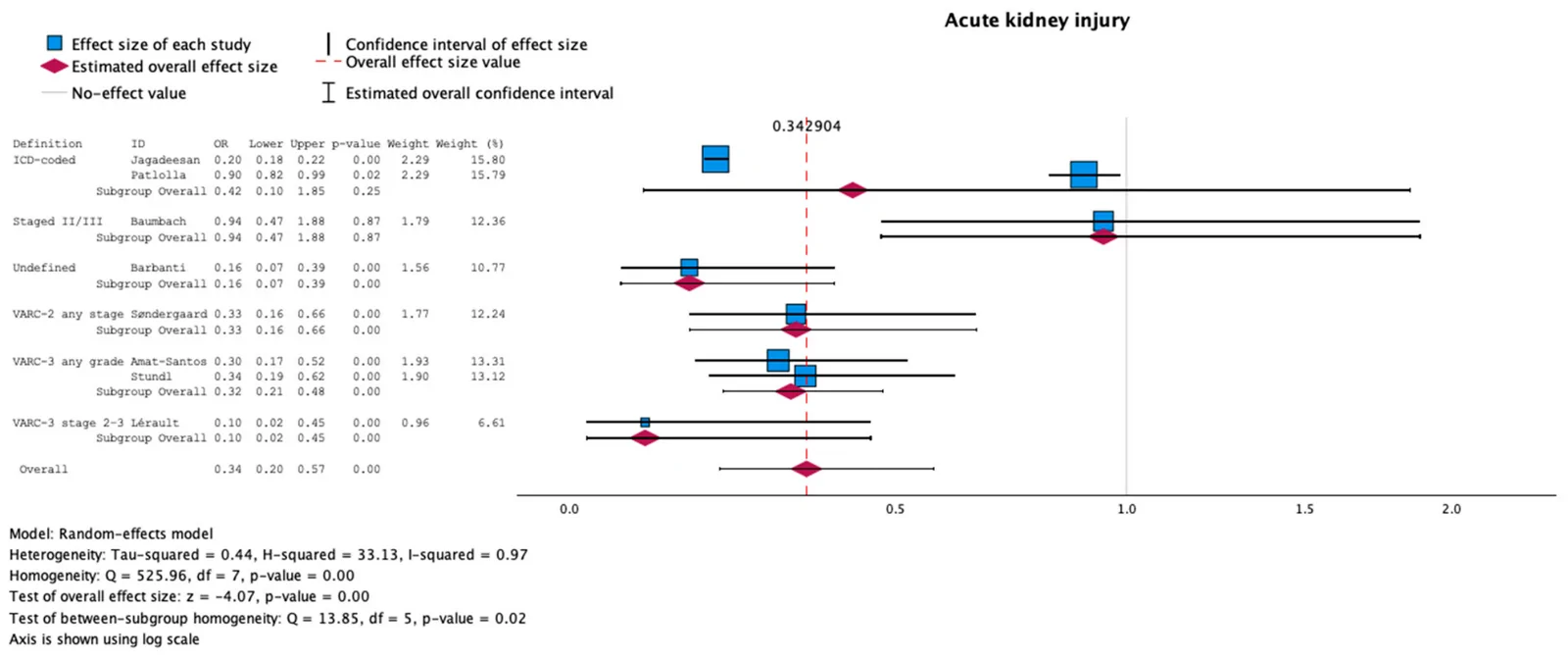
Early acute kidney injury after TAVR+PCI versus SAVR+CABG, by outcome definition. Random-effects model (REML); SAVR+CABG is the reference, and OR < 1 favours TAVR+PCI.

Eight studies reported early acute kidney injury, and all had point estimates favouring TAVR+PCI (OR 0.34, 95% CI 0.20–0.57; *p* < 0.001; Figure 9). Heterogeneity was extreme (I^2^ = 97%) and arose mainly from the two administrative databases, whose ICD-coded estimates diverged (Jagadeesan et al. [8], OR 0.20; Patlolla et al. [16], OR 0.90). Excluding them did not change the result (6 studies; OR 0.32, 95% CI 0.19–0.54; I^2^ = 64%). By definition, the association was consistent in studies using VARC any-stage criteria (3 studies; OR 0.30–0.34) but absent in the one study restricted to stage II–III injury (Baumbach et al. [34], OR 0.94, 95% CI 0.47–1.88; *p* for subgroup difference = 0.02). Small-study effects were not assessed because fewer than ten studies were available.

### 3.10. Myocardial Infarction

**Figure 10:**
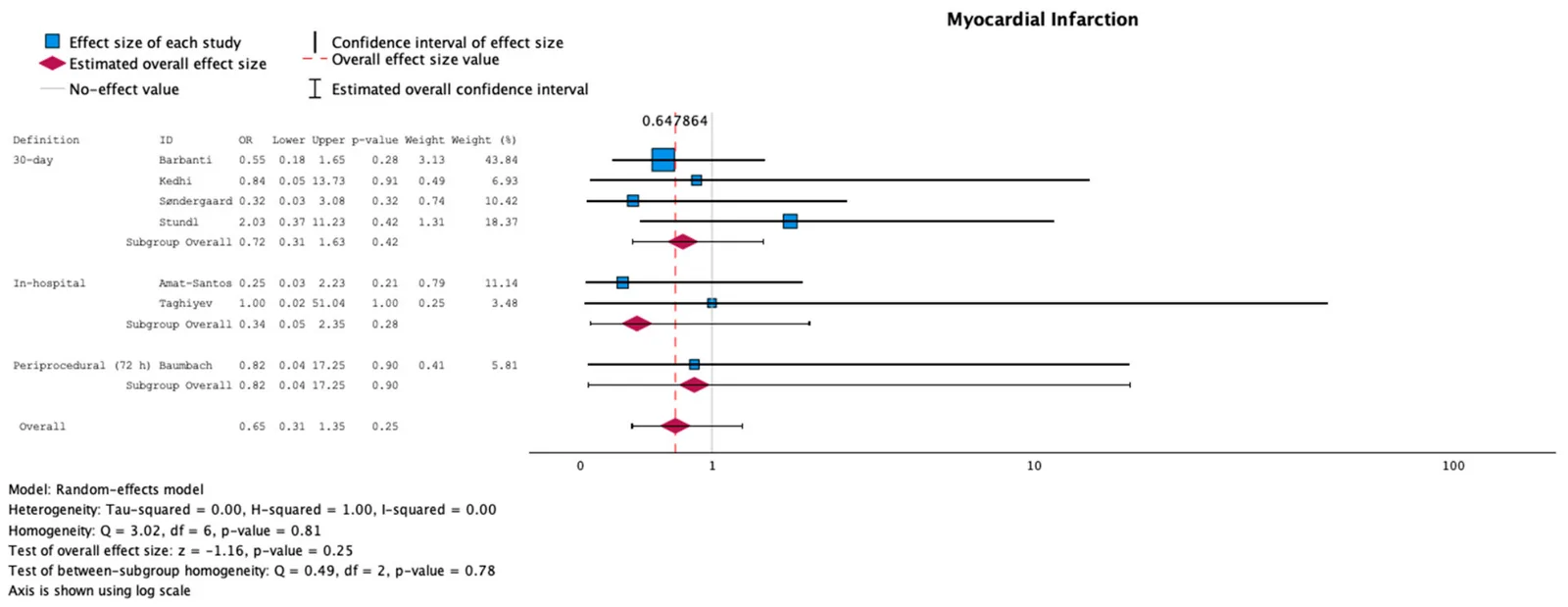
Early myocardial infarction after TAVR+PCI versus SAVR+CABG, by reporting horizon. Random-effects model (REML); SAVR+CABG is the reference, and OR < 1 favours TAVR+PCI.

Seven studies reported early myocardial infarction; one recorded no events in either arm and was not pooled. Events were sparse (12 with TAVR+PCI and 21 with SAVR+CABG), and the difference was not significant (6 studies; OR 0.64, 95% CI 0.30–1.35; I^2^ = 0%; Figure 10). Results did not differ by reporting horizon (30-day, in-hospital or 72-h periprocedural; *p* for subgroup difference = 0.78). No administrative database reported this outcome, and small-study effects were not assessed because fewer than ten studies were available.

### 3.11. Repeat Revascularization (Exploratory)

**Figure 11:**
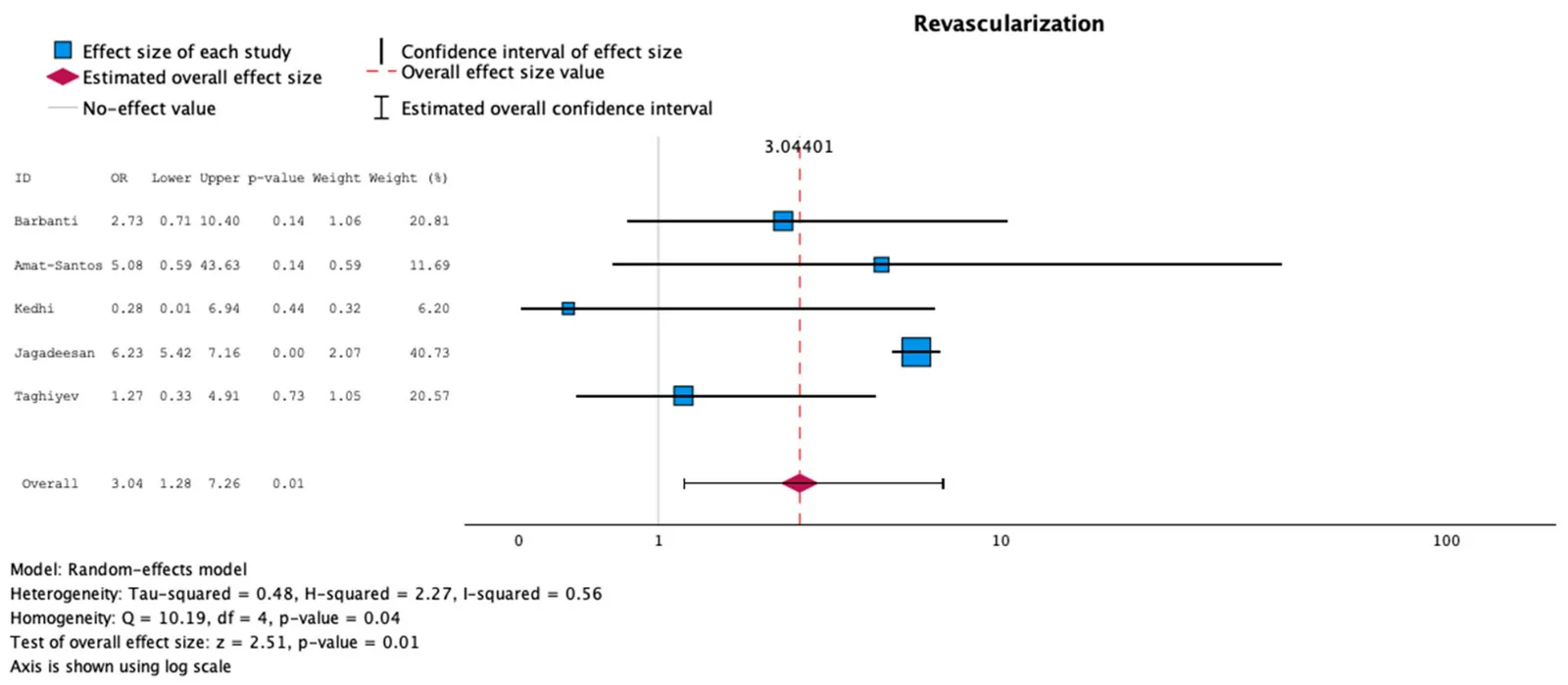
Repeat revascularization at longest follow-up after TAVR+PCI versus SAVR+CABG (exploratory). Random-effects model (REML); SAVR+CABG is the reference, and OR > 1 favours SAVR+CABG.

Five studies reported repeat revascularization at the longest available follow-up (1 to more than 5 years). TAVR+PCI was associated with more repeat revascularization than SAVR+CABG (OR 3.04, 95% CI 1.28–7.26; I^2^ = 56%; Figure 11), but the estimate was driven by the Medicare cohort (Jagadeesan et al. [8], OR 6.23, 95% CI 5.42–7.16); without it, the direction was unchanged but the difference was not significant (4 studies; OR 1.92, 95% CI 0.83–4.45; I^2^ = 0%). Because follow-up differed widely between studies and odds ratios do not account for time at risk or the competing risk of death, this analysis should be regarded as hypothesis-generating.

A summary of the GRADE results is provided in Supplementary Table S6.

## 4. Summary of Findings

We pooled 14 studies that compared TAVR+PCI with SAVR+CABG in patients with severe aortic stenosis and coronary artery disease. Most of the evidence came from observational cohorts and administrative databases. Neither strategy was better across all outcomes, and the two had different risk profiles.

TAVR+PCI was associated with lower early mortality (OR 0.60, 95% CI 0.44–0.84; about 20 fewer deaths per 1000 patients). It was also associated with less acute kidney injury (OR 0.34, 95% CI 0.20–0.57). Against this, permanent pacemaker implantation was more frequent with TAVR+PCI (OR 2.18, 95% CI 1.93–2.46; about 54 more per 1000), and so were major vascular complications (OR 3.38, 95% CI 1.65–6.89). We found no significant difference in stroke or myocardial infarction. The bleeding results were too inconsistent to interpret (I^2^ = 95%).

The early survival advantage did not last. At one year, it was no longer evident (OR 0.80, 95% CI 0.58–1.10). Over longer follow-up, mortality was higher with TAVR+PCI (HR 1.38, 95% CI 1.28–1.49), and this finding did not change when the Medicare cohort was removed (HR 1.37, 95% CI 1.17–1.61). Read together, these estimates suggest crossing hazards: TAVR+PCI carries lower procedural risk at first, and SAVR+CABG shows a survival advantage later.

Administrative databases did not determine the direction of any early outcome. They were, however, the main source of heterogeneity, because their results frequently disagreed with one another. Certainty of evidence was moderate for pacemaker implantation and low or very low for every other outcome. The randomized data were too limited to confirm or refute the observational findings.

### 4.1. Comparison with Previous Literature

Our findings agree in direction with earlier meta-analyses of this comparison and add detail to them. An early pooled analysis found no significant difference between the two strategies in 30-day mortality, stroke, myocardial infarction, or 2-year mortality. Few studies were available at that time, and the long-term estimate was highly heterogeneous [39]. A later analysis of two randomized and six observational studies reported higher mortality during follow-up with TAVR+PCI (HR 1.35), along with greater pacemaker implantation and vascular complications [13]. Two recent syntheses reached similar conclusions. One reconstructed time-to-event data from published survival curves. Both described fewer perioperative complications with TAVR+PCI and less favourable mid- to long-term outcomes [10,12]. Our long-term estimate (HR 1.38) is nearly the same as these, even though we used a different study set and excluded overlapping registry reports.

The present analysis differs from earlier ones in three respects. First, every outcome was stratified by study design and re-analysed without administrative databases. This showed that the direction of the early findings does not depend on claims data, and that most of the statistical heterogeneity comes from disagreement among the administrative databases themselves. Second, the early survival advantage of TAVR+PCI reached significance here. It had not in earlier, smaller pools. Third, in contrast to one recent synthesis [10], we found no significant reduction in stroke or myocardial infarction. The myocardial infarction analysis included only 33 events.

The pattern of an early advantage for the transcatheter strategy that fades over time is consistent with the randomized TAVR-versus-SAVR trials in lower-risk patients, where early benefits in composite endpoints narrowed with longer follow-up [40,41,42]. It is also consistent with the revascularization literature, in which CABG gave better long-term outcomes than PCI in complex multivessel disease [43,44,45]. The only dedicated randomized trial, TCW, favoured TAVR+PCI at one year. That result was driven by mortality and bleeding, but the trial was small and stopped early [6]. The excess of pacemaker implantation and vascular complications matches what every major TAVR trial has reported.

### 4.2. Strengths and Limitations

This review has four main strengths. First, we excluded overlapping reports drawn from the same registries and databases, and we tested any remaining overlap between US administrative sources by retaining one database at a time. Second, every outcome was stratified by study design and re-analysed without administrative databases. Third, the direction of each hazard ratio was verified against the published Kaplan–Meier curves. Fourth, the findings are presented with absolute effects and GRADE certainty ratings.

The limitations are substantial. Nearly all of the evidence is observational, and treatment was assigned by the Heart Team according to age, frailty and coronary anatomy. Residual confounding is therefore likely, and it probably biases the long-term comparisons against TAVR+PCI. The randomized evidence was limited to one trial subgroup and one small trial that was stopped early. Most early-outcome estimates were calculated from crude event counts.

The administrative databases identified cohorts and outcomes from ICD codes using study-specific algorithms, lacked anatomical and surgical risk data, and were the main source of heterogeneity. Outcome definitions differed across studies, particularly for bleeding, acute kidney injury and periprocedural myocardial infarction. For long-term mortality, four of the nine hazard ratios were estimated from published curves, follow-up ranged from 12 to 72 months, and one cohort carried 76% of the weight. Because the hazards were not proportional, a single hazard ratio depends on the length of follow-up.

Several outcomes had few events, and the tests for subgroup differences and small-study effects had limited power. Without patient-level data, we could not examine valve generation, staged versus concomitant PCI, or anatomical complexity. Finally, the design-stratified and exclusion analyses were added during peer review.

### 4.3. Implications for Practice and Research

These findings do not support choosing one strategy for every patient with severe aortic stenosis and coronary artery disease. The evidence is mostly of low certainty, and it points to a trade-off. TAVR+PCI carries lower early mortality and less acute kidney injury, but it leads to greater pacemaker implantation and vascular complications and a less favourable survival trajectory over subsequent years.

For patients whose main concern is procedural risk, a fully percutaneous strategy is a reasonable option. This includes patients of advanced age, frail patients, those with impaired renal function, and those with limited life expectancy. For younger patients with longer life expectancy and anatomically complex coronary disease, surgery remains difficult to displace because of its long-term survival advantage and the durability of surgical revascularization. The long-term observational comparisons probably carry residual confounding against TAVR+PCI, so the magnitude of that advantage should be interpreted with caution. Together, these findings reinforce individualized Heart Team decision-making, which should weigh anatomical complexity, the likelihood of complete revascularization, the risk of conduction disturbance, and patient preference.

Future research should prioritize adequately powered randomized trials of the two complete strategies. These trials need follow-up beyond five years, contemporary valve platforms, and stratification by SYNTAX score and surgical risk. They should report VARC-3 endpoints, completeness of revascularization, and the timing of PCI relative to TAVR, and they should use analyses suited to non-proportional hazards, such as landmark analysis or restricted mean survival time. Registries could improve comparability by adopting uniform outcome definitions. Studies based on administrative data should make their cohort identification algorithms transparent, because discordance between such databases was the main source of inconsistency in the present analysis.

## 5. Conclusions

Most of the evidence on patients with severe aortic stenosis and coronary artery disease is observational. In that evidence, TAVR+PCI was associated with lower early mortality and less acute kidney injury than SAVR+CABG. It was also associated with greater permanent pacemaker implantation, more vascular complications, and higher mortality over longer follow-up. These findings describe a trade-off between early procedural safety and long-term outcome, and they do not show that either strategy is superior. They support individualized Heart Team decisions. The certainty of evidence was low for most outcomes, and adequately powered randomized trials with long-term follow-up are needed.

## Data Availability

No new data were created or analyzed in this study. Data sharing is not applicable to this article. Data supporting the reported results were extracted from the published studies included in this meta-analysis, which are cited within the article.

## Supplementary Materials

The following supporting information can be downloaded at https://www.mdpi.com/article/10.3390/jcm15197556/s1: File S1: PRISMA 2020 checklist; Table S1: Search strategies by database; Table S2: Design, cohort, and baseline characteristics of comparative TAVR plus PCI versus SAVR plus CABG studies (Panel A, study design and analysed cohort; Panel B, baseline patient and coronary characteristics); Table S3: Definitions, assessment windows, and event adjudication in TAVR plus PCI versus SAVR plus CABG studies; Table S4: Newcastle–Ottawa Scale assessment of the included observational studies; Table S5: Cochrane RoB 2 assessment of the included randomised trials; Table S6: GRADE summary of findings: TAVR+PCI versus SAVR+CABG; Figure S1: Early all-cause mortality after TAVR+PCI versus SAVR+CABG, excluding administrative databases, by study design; Figure S2: All-cause mortality over follow-up after TAVR+PCI versus SAVR+CABG, excluding the Medicare cohort (Jagadeesan et al.), by source of the hazard ratio (reported by the original authors or estimated from Kaplan–Meier data); Figure S3: Major vascular complications after TAVR+PCI versus SAVR+CABG, excluding administrative databases, by outcome definition; Figure S4: Early stroke after TAVR+PCI versus SAVR+CABG, excluding administrative databases; Figure S5: Early major or life-threatening bleeding after TAVR+PCI versus SAVR+CABG, excluding administrative databases, by outcome definition. References [10,11,12] are cited in the Supplementary Materials.
