# Supplementary material for: Clinical Outcomes of Combined TAVR-PCI Versus SAVR-CABG in Patients with Aortic Stenosis and Coronary Artery Disease: A Systematic Review and Meta-Analysis
Source: J Clin Med. 2026 Sep 29;15(19):7556. doi: 10.3390/jcm15197556 (PMC13665907; doi:10.3390/jcm15197556)
Supplement: Supplementary file 1 [file jcm-15-07556-s001.zip › jcm-4547911-supplementary.pdf]

**Supplementary Material**

**Table S1. Search strategies by database.** Searches run from inception to July 2026; no language or date filters.

| Database | Search strategy | Records |
|---|---|---|
| **PubMed / MEDLINE** | #1 "Aortic Valve Stenosis"[Mesh] OR "aortic stenosis"[tiab] OR "aortic valve stenosis"[tiab]<br>#2 "Coronary Artery Disease"[Mesh] OR "Myocardial Revascularization"[Mesh] OR "coronary artery disease"[tiab] OR "coronary disease"[tiab] OR "coronary revascularization"[tiab] OR "coronary revascularisation"[tiab]<br>#3 "Transcatheter Aortic Valve Replacement"[Mesh] OR "transcatheter aortic valve"[tiab] OR TAVR[tiab] OR TAVI[tiab]<br>#4 "Percutaneous Coronary Intervention"[Mesh] OR "percutaneous coronary intervention"[tiab] OR PCI[tiab] OR stent*[tiab]<br>#5 "Heart Valve Prosthesis Implantation"[Mesh] OR "surgical aortic valve replacement"[tiab] OR SAVR[tiab] OR "aortic valve replacement"[tiab]<br>#6 "Coronary Artery Bypass"[Mesh] OR "coronary artery bypass"[tiab] OR CABG[tiab] OR "bypass graft*"[tiab]<br>#7 #1 AND #2 AND (#3 OR #4) AND (#5 OR #6) | 612 |
| **Embase (Ovid)** | 1. exp aortic valve stenosis/ or (aortic stenosis or aortic valve stenosis).ti,ab.<br>2. exp coronary artery disease/ or exp coronary artery bypass graft/ or exp percutaneous coronary intervention/ or (coronary artery disease or coronary revascularization or coronary revascularisation).ti,ab.<br>3. exp transcatheter aortic valve implantation/ or (transcatheter aortic valve or TAVR or TAVI).ti,ab.<br>4. (percutaneous coronary intervention or PCI or stent*).ti,ab.<br>5. exp aortic valve replacement/ or (surgical aortic valve replacement or SAVR or aortic valve replacement).ti,ab.<br>6. (coronary artery bypass or CABG or bypass graft*).ti,ab.<br>7. 1 and 2 and (3 or 4) and (5 or 6)<br>8. limit 7 to exclude medline journals | 688 |

| Database | Search strategy | Records |
|---|---|---|
| **Cochrane CENTRAL** | #1 MeSH descriptor: [Aortic Valve Stenosis] explode all trees<br>#2 (aortic stenosis OR aortic valve stenosis):ti,ab,kw<br>#3 MeSH descriptor: [Coronary Artery Disease] explode all trees<br>#4 (coronary artery disease OR coronary revascularization OR coronary revascularisation):ti,ab,kw<br>#5 (transcatheter aortic valve OR TAVR OR TAVI):ti,ab,kw<br>#6 (percutaneous coronary intervention OR PCI):ti,ab,kw<br>#7 (surgical aortic valve replacement OR SAVR OR aortic valve replacement):ti,ab,kw<br>#8 (coronary artery bypass OR CABG):ti,ab,kw<br>#9 (#1 OR #2) AND (#3 OR #4) AND (#5 OR #6) AND (#7 OR #8) | 341 |
| **Web of Science Core Collection** | TS=("aortic stenosis" OR "aortic valve stenosis") AND TS=("coronary artery disease" OR "coronary revascularization" OR "coronary revascularisation") AND TS=("transcatheter aortic valve" OR TAVR OR TAVI OR "percutaneous coronary intervention" OR PCI) AND TS=("surgical aortic valve replacement" OR SAVR OR "aortic valve replacement" OR "coronary artery bypass" OR CABG) | 197 |
| **ClinicalTrials.gov** | Condition: aortic stenosis AND coronary artery disease. Intervention/treatment: TAVR OR TAVI OR transcatheter aortic valve. Other terms: CABG OR SAVR OR surgical. Study type: all; status: all. Results reviewed for completed trials with posted or published results. | 24 |
| **Citation searching** | Reference lists of all included studies and of Fang 2026 [10], Baudo 2026 [11], and Emara 2026 [12]. | 3 |

Footnote: Searches were last run in July 2026. Embase line 8 removes MEDLINE-indexed records to reduce duplication before de-duplication in Zotero. Truncation (*) captures plural and variant forms.

# Study Characteristics for Combined Aortic Valve and Coronary Revascularization Strategies

**Table S2. Design, cohort, and baseline characteristics of comparative TAVR plus PCI versus SAVR plus CABG studies**

## Panel A Study design and analysed cohort

| First author, year | Country and centres | Data source and design | Enrollment period | Adjustment method and cohort analysed | n TAVR + PCI / n SAVR + CABG | PCI timing | Longest reported follow-up |
|---|---|---|---|---|---|---|---|
| **Wendt, 2013** | Germany; West-German Heart Center Essen; single centre (1) | Prospective institutional database; retrospective nonrandomized observational analysis | Not reported | Propensity-score adjustment using 10 strata plus multivariable logistic regression; unmatched, unweighted cohort | 59 / 184 | All PCI staged before TAVR within 12 months; mean interval 82 ± 93 days (range 3-340) | 6 years |
| **Barbanti, 2018** | Italy; national multicentre study (93 centres) | OBSERVANT prospective registry; post-hoc observational analysis; follow-up linked to national hospital-discharge and tax registries | Dec 2010 to Jun 2012 | 1:1 nearest-neighbour propensity-score matching plus doubly robust multivariable adjustment; matched, unweighted cohort | 236 / 236 | Before TAVR (<6 months), 217/236 (92%); concomitant, 19/236 (8%) | 3 years |
| **Baumbach, 2019** | Germany; Robert Bosch Hospital, Stuttgart; single centre (1) | Prospective institutional observational registry | Jan 2008 to Oct 2015 | Multivariable Cox regression; no propensity method; unmatched, unweighted cohort | 112 / 464 | All PCI staged before TAVR within 12 months; interval not reported | Mean 19.0 ± 14.8 / 25.0 ± 27.1 months; 12-month outcomes reported |
| **Søndergaard, 2019** | United States, Canada, and Europe; multicentre (87 centres) | SURTAVI prospective randomized controlled trial (NCT01586910) | 19 Jun 2012 to 30 Jun 2016 | 1:1 randomization, stratified by centre and need for revascularization; modified intention-to-treat; unweighted | 169 / 163 | As-treated TAVR + PCI cohort (n = 128): staged at least 7 days before TAVR, 76 (59.4%); concomitant, 52 (40.6%) | 2 years |
| **Alperi, 2021** | North America and Europe; 14 TAVR centres plus 1 North American SAVR centre | Dedicated multicentre institutional databases with prospectively collected data; retrospective comparative analysis | 2007 to 2019 | 1:1 nearest-neighbour propensity-score matching without replacement (caliper 0.2), followed by Cox regression; matched, unweighted cohort | 156 / 156 | PCI within 3 months before or concomitant with TAVR; median interval 34 days (IQR 11-71); concomitant proportion not reported | Median 3 years (IQR 1-6); Kaplan-Meier estimates to 5 years |
| **Patlolla, 2022** | United States; nationwide multicentre sample; number of hospitals not reported | HCUP National (Nationwide) Inpatient Sample; retrospective administrative admission cohort | Jan 2012 to Dec 2017 | NIS discharge weighting and multivariable hierarchical logistic regression; unmatched, weighted admission-level cohort | 4,240 / 97,955 | Same hospitalization: before TAVR, 1,515/3,775 (40.1%); same day, 2,260/3,775 (59.9%). PCI after TAVR was excluded | In-hospital only |
| **Elderia, 2023** | Germany; University of Cologne Heart Center; single centre (1) | Institutional medical records; retrospective consecutive cohort | From 2017; end date not reported | 1:1 propensity-score matching; matched, unweighted cohort | 101 / 101 | All PCI staged before TAVR within 6 months; median interval 43 days (IQR 1-171) | 30 days |
| **Lérault, 2023** | France; Reims University Hospital; single centre (1) | Consecutive institutional cohort; retrospective medical-record review | 2013 to 2020 | Propensity-score inverse probability treatment weighting, followed by weighted univariable and multivariable Cox regression; unmatched, weighted analyses | 150 / 91 | All PCI staged before TAVR; no simultaneous PCI and TAVR | Median 27 months (IQR 14-39); Kaplan-Meier curves extend to 4 years |
| **Ullah, 2023** | United States; nationwide multicentre sample from 22 states; number of hospitals not reported | HCUP Nationwide Readmissions Database (NRD); retrospective administrative cohort | Sep 2015 to Nov 2019 | One-to-many nearest-neighbour propensity-score matching without replacement plus multivariable regression for residual imbalance; matched/weighted national cohort | 5,358 / 6,003 | PCI during the TAVR admission or within 3 months before TAVR; same-day 2,593 and different-day 2,306 among cases with timing reported | 30 days |

| First author, year | Country and centres | Data source and design | Enrollment period | Adjustment method and cohort analysed | n TAVR + PCI / n SAVR + CABG | PCI timing | Longest reported follow-up |
|---|---|---|---|---|---|---|---|
| **Amat-Santos, 2024** | Spain; national multicentre registry (14 tertiary centres) | Dedicated multicentre registry; retrospective consecutive cohort | Jan 2018 to Dec 2022 | 1:1 nearest-neighbour propensity-score matching without replacement (caliper 0.15), followed by Cox regression; matched, unweighted cohort | 254 / 254 | Before TAVR, 50.9% (mean 24.1 ± 5.2 days); concomitant, 16.3%; after TAVR, 32.8% (mean 37.4 ± 7.4 days) | 1 year |
| **Kedhi, 2024** | Ten European countries; international multicentre trial (18 tertiary centres) | TCW prospective, open-label, noninferiority randomized controlled trial (NCT03424941) | 31 May 2018 to 30 Jun 2023 | 1:1 site-stratified block randomization; primary and safety analyses by protocol-defined intention-to-treat; unweighted | 91 / 77 analysed (81 randomized) | FFR-guided PCI simultaneous with TAVR or staged before TAVR; staged TAVR no more than 40 days after PCI; proportions not reported | 1 year |
| **Jagadeesan, 2025** | United States; national Medicare cohort; number of centres not reported | Centers for Medicare & Medicaid Services (CMS) inpatient claims; retrospective cohort | Jan 2018 to Dec 2022 | Inverse-probability propensity weighting plus doubly robust multilevel regression, Cox, and Fine-Gray models; unmatched, weighted cohort | 17,413 / 20,409 | Within 3 months before TAVR, 14,500 (83.3%); same admission, 2,337 (13.4%); within 3 months after, 576 (3.3%) | 5 years |
| **Taghiyev, 2025** | Germany; University Hospital Giessen tertiary referral centre; single centre (1) | Consecutive institutional cohort; retrospective analysis with prospectively collected follow-up | Jan 2010 to Dec 2020 | 1:1 propensity-score matching without replacement using age, LVEF, SYNTAX I, STS, and EuroSCORE; matched, unweighted cohort | 76 / 76 | PCI within 90 days before TAVR; median interval 12 days (IQR 6-27) | Median 5.3 years (IQR 3.3-8.99); Kaplan-Meier curves extend to 10 years |
| **Stundl, 2026** | Germany; multicentre registry (3 high-volume heart centres) | Multicentre institutional database/registry; retrospective cohort | 2012 to 2020 | 1:1 nearest-neighbour propensity-score matching; matched, unweighted cohort | 154 / 154 | All PCI staged before TAVR; median interval 29.5 days (IQR 7-43.3) | 3 years |

**Panel B Baseline patient and coronary characteristics**

| First author, year | Mean age, years | Female, % | Surgical risk score | CAD complexity |
|---|---|---|---|---|
| **Wendt, 2013** | 80 ± 6 / 75 ± 6 | 55.9 / 54.3 | STS-PROM: 16.7 ± 10.5% / 13.1 ± 8.7%; logistic EuroSCORE: 27.5 ± 16.3% / 18.1 ± 13.8% | 1-vessel: 55.9% / 34.8%; 2-vessel: 39.0% / 34.2%; 3-vessel: 5.1% / 31.0%; SYNTAX and left-main disease not reported |
| **Barbanti, 2018** | 80.7 ± 6.2 / 80.5 ± 5.2 | 45.8 / 44.1 | EuroSCORE II: 7.8 ± 8.8% / 7.8 ± 6.0% | Stable CAD with >50% stenosis required; SYNTAX score, multivessel disease, and left-main disease not reported |
| **Baumbach, 2019** | 81.3 ± 5.7 / 78.7 ± 3.1 | 34.8 / 36.2 | Logistic EuroSCORE I: 36.1 ± 18.9% / 21.0 ± 17.5% | Multivessel disease: 74.1% / 77.4%; SYNTAX and left-main disease not reported |
| **Søndergaard, 2019** | Not reported by randomized arm; overall revascularization cohort: 79.5 ± 5.9 | Not reported by randomized arm; overall: 34.9 (116/332) | STS-PROM not reported by randomized arm; overall: 4.8 ± 1.7% | SYNTAX: 8.1 ± 5.6 / 8.5 ± 5.6; multivessel disease: 60.4% / 62.6%; left main: 4.1% / 7.4%. Patients with multivessel SYNTAX >22 and an unprotected left coronary artery were excluded |
| **Alperi, 2021** | 79.5 ± 8.0 / 79.0 ± 6.7 | 33.5 / 36.0* | STS-PROM: 5.8 ± 5.1% / 5.7 ± 4.3% | SYNTAX: 26.3 ± 8.1 / 26.9 ± 7.5; left main: 55.8% / 57.1%; complex CAD (unprotected left main or SYNTAX >22) required |
| **Patlolla, 2022** | 80.8 ± 8.2 / 72.6 ± 8.9 | 46.3 / 26.8 | Not reported | SYNTAX score, multivessel disease, and left-main disease not reported in NIS |
| **Elderia, 2023** | Means not reported; medians: 78.5 (IQR 74.5-82.0) / 77.2 (74.8-80.2) | 31.7 / 31.7 | EuroSCORE II: 7.6 ± 5.1% / 6.2 ± 2.7% | 3-vessel disease: 38.6% / 38.6%; left main: 28.7% / 36.6%; SYNTAX not measured |
| **Lérault, 2023** | 83.5 / 71.8 (means); medians: 84 (81-86) / 73 (67-77) | 40.7 / 26.4 | Logistic EuroSCORE: 11.1% / 5.7% (means); medians: 8.5 (5.1-12.6) / 4.5 (3.5-7.0) | SYNTAX: 13.1 / 17.2 (means); medians: 10 (6-18) / 13.5 (8-22.5). Multivessel disease: 51.3% / 69.3%; left main: 8.7% / 17.6% |
| **Ullah, 2023** | 79.6 / 70.2 (crude means; matched values not reported) | 44.0 / 24.9 (crude; matched table internally inconsistent)* | Not reported | Matched revascularization: single artery 79.2% / 55.4%; two arteries 16.9% / 31.7%; three arteries 3.1% / 10.7%; four arteries 0.6% / 2.2%; SYNTAX and left-main disease not reported |
| **Amat-Santos, 2024** | 77.9 ± 5.7 / 77.4 ± 5.3 | 34.6 / 31.5† | STS-PROM, median (IQR): 3.4% (2.4-5.3) / 3.0% (2.1-3.9); EuroSCORE II: 2.7% (1.7-4.7) / 2.9% (2.1-4.6) | SYNTAX: 26.4 ± 7.4 / 27.2 ± 8.1; three-vessel or left-main disease: 23.2% / 38.2% |
| **Kedhi, 2024** | 76.3 ± 3.7 / 76.6 ± 4.1 | 35 / 27 | STS-PROM, median (IQR): 2.3% (1.7-3.6) / 2.6% (1.7-3.9); EuroSCORE II: 2.3% (1.7-3.3) / 2.8% (1.9-3.9) | SYNTAX, median (IQR): 13.0 (10.0-17.0) / 12.0 (8.0-17.0); ≥2-vessel disease: 76% / 76%; left main: 2% / 4% |
| **Jagadeesan, 2025** | 77.1 ± 5.91 / 72.7 ± 5.99 (unweighted) | 35.7 / 22.9 (unweighted) | STS-PROM and EuroSCORE not available in CMS claims | Multivessel revascularization: 9.6% / 46.2% (unweighted); weighted baseline variables were balanced; anatomy, SYNTAX, and left-main disease unavailable |
| **Taghiyev, 2025** | 78.8 ± 3.5 / 78.5 ± 3.9 | 38 / 28 | STS-PROM: 3.0 ± 1.7% / 3.4 ± 1.2%; EuroSCORE II: 6.89 ± 4.3% / 7.41 ± 4.7% | SYNTAX I: 24.05 ± 8.1 / 27.01 ± 11.3; complex CAD (SYNTAX >22 or significant untreated left-main disease) was required |
| **Stundl, 2026** | 79.7 ± 5.2 / 78.5 ± 5.0 | 40.3 / 42.9 | STS-PROM: 4.5 ± 2.2% / 4.2 ± 2.1%; EuroSCORE II: 6.0 ± 3.0% / 6.2 ± 2.9%; logistic EuroSCORE: 14.0 ± 2.7% / 13.7 ± 2.7% | SYNTAX: 16.2 ± 10.9 / 22.2 ± 13.5; 3-vessel disease: 40.3% / 51.3%; left main: 20.8% / 29.2% |

**Notes.** Panel B uses the adjusted or randomized cohort where arm-specific values were available; exceptions are labelled. Patlolla sample sizes are NIS-weighted estimates. Søndergaard arm-specific age, sex, and STS-PROM were not provided; overall randomized-cohort values are shown. Baumbach follow-up was available for 45/112 TAVR + PCI and

247/464 SAVR + CABG patients. Kedhi was published in 2024 despite the supplied file name indicating 2025; four patients randomized to surgery died before treatment and were excluded under the trial's protocol-defined intention-to-treat analysis, leaving 77 analysed.

**Data-quality flags.** *The Alperi and Ullah published matched tables contain internally inconsistent sex counts/percentages; Alperi's reported percentages and Ullah's crude values are retained and labelled. †The Amat-Santos matched row labelled female corresponds numerically to male counts; female percentages shown here are the complements. National administrative cohorts (NIS, NRD, and CMS) may contain overlapping US hospitalizations or beneficiaries and cannot be deduplicated across studies from the reports.

**Abbreviations.** CABG, coronary artery bypass grafting; CAD, coronary artery disease; CMS, Centers for Medicare & Medicaid Services; FFR, fractional flow reserve; HCUP, Healthcare Cost and Utilization Project; IQR, interquartile range; IPTW/IPW, inverse probability treatment weighting; LVEF, left ventricular ejection fraction; NIS, National (Nationwide) Inpatient Sample; NRD, Nationwide Readmissions Database; PCI, percutaneous coronary intervention; PSM, propensity-score matching; SAVR, surgical aortic valve replacement; STS-PROM, Society of Thoracic Surgeons Predicted Risk of Mortality; TAVR/TAVI, transcatheter aortic valve replacement/implantation.

Outcome Definitions and Ascertainment Across Comparative Studies

**Table S3. Definitions, assessment windows, and event adjudication in TAVR plus PCI versus SAVR plus CABG studies**

| First author, year | Definition set used | Early mortality | Mid-term mortality horizon | Stroke definition and neurologist adjudication | AKI definition | MI definition and horizon | Revascularization reported and horizon | Pacemaker horizon | Independent event adjudication | Horizon used in this meta-analysis |
|---|---|---|---|---|---|---|---|---|---|---|
| **Wendt, 2013** | Institutional definitions; EuroSCORE definitions used for selected comorbidities | 30-day all-cause death, labelled in-hospital/perioperative mortality; tracked from the institutional database or active follow-up | All-cause survival at 2, 4, and 6 years | Stroke at 30 days; specific definition not reported; neurologist adjudication not reported | Not reported | Not reported as an outcome | Yes; cardiac valve or vascular reintervention evaluated through follow-up to 6 years; definition not reported | Early/periprocedural; exact window not explicitly stated | No independent committee reported | Mortality: 30 days and 6 years; stroke: 30 days; pacemaker: early. AKI and MI not used. Revascularization not used because no surgical-arm count was reported. |
| **Barbanti, 2018** | OBSERVANT registry plus administrative record linkage; explicitly not VARC | 30-day all-cause mortality | 1, 2, and 3 years | All stroke from hospital-discharge records at 30 days and 1, 2, and 3 years; no neurologist adjudication reported | Periprocedural/in-hospital AKI; criteria not defined | Acute MI from hospital-discharge records; 30 days and 1, 2, and 3 years; coding algorithm not specified in the article | Yes; unplanned revascularization at 30 days and 1, 2, and 3 years, defined as CABG/PCI at least 30 days after index treatment or for acute MI at any time | In-hospital | No event committee reported; independent observers performed registry data-quality monitoring only | Mortality, stroke, MI, and revascularization: 30 days and 3 years. AKI and pacemaker: in-hospital. |
| **Baumbach, 2019** | Institutional definitions | Intraoperative, in-hospital, and 30-day all-cause mortality | 12-month survival; mean available follow-up approximately 19/25 months | Stroke/TIA within 72 hours; definition not reported; no neurologist adjudication reported | AKI stage II/III reported; diagnostic criteria not specified. CKD was defined using KDIGO/KDOQI | Periprocedural MI within 72 hours; definition not reported | No systematic repeat coronary revascularization outcome; 12-month rehospitalization was reported | Postoperative/30-day | No independent committee reported | Mortality: in-hospital/30 days and 1 year; stroke and MI: peri-procedural; AKI and pacemaker: 30 days. Revascularization not used. |
| **Søndergaard, 2019** | VARC-2 and other prespecified standardized trial definitions | 30-day all-cause mortality | 2 years | All stroke and disabling stroke at 30 days and 2 years; every patient examined by a neurologist or trained stroke specialist and neurologic events adjudicated by a neurologist | VARC-2 AKI stages 1-3 at 30 days | VARC-2 MI at 30 days and 2 years | No reliable postprocedure coronary revascularization outcome; index completeness reported. Aortic-valve reintervention reported at 2 years | 30 days and 2 years | Yes; independent clinical events committee | Mortality: 30 days and 2 years; stroke: 30 days and 2 years; AKI: 30 days; MI: 2 years; pacemaker: 30 days and 2 years. Coronary revascularization not extractable; aortic-valve reintervention was not used. |
| **Alperi, 2021** | VARC-2 plus acute coronary syndrome guideline definitions | Periprocedural/30-day event period; 30-day landmark analysis reported | Up to 5 years; median follow-up 3 years | All stroke; disabling status and neurologist adjudication not reported | Not reported as an outcome | Nonprocedural MI through 5 years; VARC-2/ACS definitions; type 2 NSTEMI excluded | Yes; new coronary revascularization through 5 years | Not reported | No; authors explicitly state there was no independent event-adjudication committee | Mortality, stroke, MI, and coronary revascularization: 5 years. AKI and pacemaker not used. |

| First author, year | Definition set used | Early mortality | Mid-term mortality horizon | Stroke definition and neurologist adjudication | AKI definition | MI definition and horizon | Revascularization reported and horizon | Pacemaker horizon | Independent event adjudication | Horizon used in this meta-analysis |
|---|---|---|---|---|---|---|---|---|---|---|
| **Patlolla, 2022** | ICD-9-CM and ICD-10-CM diagnosis and procedure codes in HCUP-NIS | In-hospital mortality only | Not reported | Acute ischemic stroke during index hospitalization, ICD-coded; no neurologist adjudication | ICD-coded AKI during index hospitalization; no stage or clinical criteria available | Not reported as an outcome | No repeat revascularization outcome; only index CABG/PCI identified by procedure codes | In-hospital | No; administrative claims data | Mortality, stroke, AKI, and pacemaker: in-hospital. MI and repeat revascularization not used. |
| **Elderia, 2023** | VARC criteria cited, including VARC-2 and VARC-3 references; institutional ascertainment | In-hospital complications and 30-day all-cause mortality | Not reported | Any new cerebral neurologic deficit during the primary stay with CT/MRI evidence; assessed by a neurologist; disabling status not reported | Periprocedural acute renal failure/dialysis; VARC framework stated but stage and criteria not reported | Type 5 MI associated with surgery, AVR, or TAVI; periprocedural/in-hospital | Yes; repeat revascularization assessed at 30 days (none observed) | 30 days | No independent committee reported; neurologist assessment was institutional | Mortality, stroke, and pacemaker: 30 days; MI: in-hospital; revascularization: 30 days. AKI not used because only postoperative creatinine clearance/dialysis was reported, without an AKI definition. |
| **Lérault, 2023** | VARC-3 for early outcomes; institutional records/physician contact for late outcomes | 30-day mortality | Median 27 months; time-to-event curves extend to 4 years | All stroke at 30 days and during long-term follow-up; disabling status and neurologist adjudication not reported | VARC-3 AKI stage 2/3, defined as at least 200% serum-creatinine increase; 30 days | Long-term MI through follow-up to 4 years; specific MI definition not stated | No repeat revascularization outcome; procedural completeness reported only | 30 days | No independent committee reported | Mortality, stroke, AKI, and pacemaker: 30 days; late mortality, stroke, and MI: longest reported follow-up (median 27 months; curves to 4 years). Revascularization not used. |
| **Ullah, 2023** | ICD-10-CM diagnosis/procedure codes in HCUP-NRD; detailed code definitions in supplement | Index in-hospital mortality; 30-day deaths captured only when occurring during readmission | Not reported | All ICD-coded stroke during index admission and 30-day readmission; no disabling classification or neurologist adjudication | Not reported as a post-procedural outcome | MI type unavailable; MI not reported as a separate endpoint, and ischemic heart disease was used as a readmission category | No repeat revascularization endpoint; index procedure and number of treated vessels reported | Index hospitalization and 30-day readmission | No; administrative claims data | Mortality, stroke, and pacemaker: index admission and 30-day readmission. AKI, MI, and repeat revascularization not used. |
| **Amat-Santos, 2024** | VARC-3 for TAVR plus PCI events; analogous institutional criteria for surgical technical success | Procedural, in-hospital, and 30-day mortality. In-hospital death was used for the early meta-analysis (TAVR + PCI 6/254; SAVR + CABG 16/254) because the supplementary 30-day row reports inconsistent counts (4/254 and 2/254, respectively) | 1 year | All stroke, defined as acute ischemic/hemorrhagic neurologic dysfunction persisting at least 24 hours or until death; neurologist adjudication not reported | VARC-3 AKI, reported in-hospital; stage not provided | Fourth Universal Definition; nonprocedural MI reported at 30 days and 1 year | Yes; new coronary revascularization at 30 days and 1 year | In-hospital | No; authors explicitly state that independent adjudication was not performed | Early mortality: in-hospital; stroke, AKI, and pacemaker: in-hospital; mortality, stroke, MI, and coronary revascularization: 1 year. |
| **Kedhi, 2024** | VARC-2 endpoint definitions | 30-day all-cause and cardiovascular mortality | 1 year | Stroke/TIA, all stroke, disabling and nondisabling stroke at 30 days and 1 year; no neurologist specified on the event committee | Not reported | VARC-2 any, periprocedural, and spontaneous MI at 30 days and 1 year | Yes; any revascularization and clinically driven target-vessel revascularization at 30 days and 1 year | 30 days and 1 year | Yes; independent clinical event committee of two interventional cardiologists and two cardiac surgeons; committee was unblinded | Mortality, stroke, MI, coronary revascularization, and pacemaker: 30 days and 1 year. AKI not used. |
| **Jagadeesan, 2025** | ICD-10 diagnosis/procedure codes in CMS inpatient claims | In-hospital mortality | Up to 5 years | All stroke: index event if not present on admission or a later admission with stroke as primary diagnosis; no disabling classification or neurologist adjudication | ICD-coded AKI during index hospitalization; no clinical stage | Later admission with MI as primary diagnosis; followed up to 5 years | Yes; coronary and valve reintervention through 5 years | In-hospital | No; administrative claims data | Mortality, AKI, and pacemaker: in-hospital; mortality, stroke, MI, and coronary reintervention: 5 years. |

| First author, year | Definition set used | Early mortality | Mid-term mortality horizon | Stroke definition and neurologist adjudication | AKI definition | MI definition and horizon | Revascularization reported and horizon | Pacemaker horizon | Independent event adjudication | Horizon used in this meta-analysis |
|---|---|---|---|---|---|---|---|---|---|---|
| **Taghiyev, 2025** | VARC-3 for postoperative outcomes | In-hospital and 30-day all-cause mortality | Median 5.3 years; Kaplan-Meier follow-up extends to 10 years | Stroke/ischemic stroke during follow-up; disabling status and neurologist adjudication not reported | Dialysis-requiring renal failure reported periprocedurally; VARC-3 framework stated but stage not reported | VARC-3 acute periprocedural MI and MI during follow-up to approximately 10 years | Yes; repeat coronary revascularization during follow-up to approximately 10 years | Periprocedural and mid-term follow-up | No; adverse events were self-adjudicated | Mortality: 30 days and long-term follow-up (median 5.3 years); stroke, MI, revascularization, and pacemaker: long-term follow-up. AKI not used because only dialysis-requiring renal failure was reported. |
| **Stundl, 2026** | VARC-3 for 30-day secondary outcomes | 30-day all-cause mortality | 6 months and 1, 2, and 3 years | All stroke at 30 days; disabling status and neurologist adjudication not reported | VARC-3 AKI grades 1-3 at 30 days | VARC-3 MI at 30 days | No repeat revascularization outcome reported | 30 days | No independent committee reported | Mortality: 30 days and 3 years; stroke, AKI, MI, and pacemaker: 30 days. Revascularization not used. |

**Interpretive note.** For administrative datasets, a reported 30-day readmission event is not equivalent to complete 30-day clinical follow-up because out-of-hospital events and deaths without readmission may be missed. Neurologist adjudication was recorded as present only when the article explicitly described neurologist assessment or adjudication.

**Meta-analysis horizon.** The final column records the outcome-specific timepoint entered into the quantitative synthesis, rather than every timepoint available in the source report. Not used identifies outcomes excluded because the event was not defined, was not post-procedural, lacked arm-specific counts, or represented aortic-valve rather than coronary reintervention.

**Abbreviations.** AKI, acute kidney injury; CABG, coronary artery bypass grafting; CEC, clinical events committee; CMS, Centers for Medicare & Medicaid Services; CT, computed tomography; HCUP, Healthcare Cost and Utilization Project; ICD, International Classification of Diseases; KDIGO, Kidney Disease Improving Global Outcomes; KDOQI, Kidney Disease Outcomes Quality Initiative; MI, myocardial infarction; NIS, National Inpatient Sample; NRD, Nationwide Readmissions Database; PCI, percutaneous coronary intervention; SAVR, surgical aortic valve replacement; TAVR/TAVI, transcatheter aortic valve replacement/implantation; TIA, transient ischemic attack; VARC, Valve Academic Research Consortium.

# Table S4. Newcastle–Ottawa Scale assessment of the included observational studies

## Wendt 2013 (Ann Thorac Surg 95:599)

| Item | Criterion | Score | Justification (page / section) |
|---|---|---|---|
| **S1** | Representativeness of exposed cohort (TAVR+PCI) | ★ | 243 consecutive high-risk patients from a prospective institutional database, single centre (Methods p.600). |
| **S2** | Selection of non-exposed cohort (SAVR+CABG) | ★ | AVR+CABG patients from the same institution and period (2006–2011), same database (Methods p.600). |
| **S3** | Ascertainment of exposure | ★ | Procedures from the prospective institutional database (Methods p.600). |

| Item | Criterion | Score | Justification (page / section) |
|---|---|---|---|
| **S4** | Outcome not present at start | ★ | Outcomes are post-procedural by definition; in-hospital mortality defined as all deaths within 30 days (Methods p.600). |
| **C1** | Comparability: age + risk score / comorbidity index | 0 | Main comparisons are crude; the propensity-score-stratified analysis (10 strata; LVEF, pulmonary hypertension, renal insufficiency, STS, EuroSCORE, prior surgery) is reported for in-hospital mortality only (OR 0.62, Results p.601) — scored 0 per protocol, as for all studies whose adjustment is limited to mortality. |
| **C2** | Comparability: CAD complexity or LV function | 0 | 0 — no adjusted comparison for the other outcomes (see C1). |
| **O1** | Assessment of outcome | 0 | Outcomes from the institutional database; no VARC definitions, no adjudication, and stroke/pacemaker definitions not reported (Methods p.600) — not reported. |
| **O2** | Follow-up long enough (≥30 days) | ★ | 30-day outcomes and long-term survival to 6 years (Results p.601, Fig 1 p.602). |
| **O3** | Adequacy of follow-up (≥90%) | ★ | 'Completeness of follow-up was 100%' (Methods p.600). |
| **Total** | Selection 4 / Comparability 0 / Outcome 2 | **6/9** | **Quality: Poor** |

## Barbanti 2018 (Int J Cardiol 270:102)

| Item | Criterion | Score | Justification (page / section) |
|---|---|---|---|
| **S1** | Representativeness of exposed cohort (TAVR+PCI) | ★ | OBSERVANT: national prospective registry enrolling consecutive patients undergoing TAVI or SAVR (Methods 2.1, p.102–103). |

| Item | Criterion | Score | Justification (page / section) |
|---|---|---|---|
| **S2** | Selection of non-exposed cohort (SAVR+CABG) | ★ | SAVR+CABG patients from the same registry and period (2010–2012), post-hoc analysis with identical inclusion criteria (Methods p.103). |
| **S3** | Ascertainment of exposure | ★ | Registry entries with on-site monitoring against charts (Methods 2.1, p.103). |
| **S4** | Outcome not present at start | ★ | Outcomes are post-index-procedure by definition; unplanned revascularization defined as ≥30 d after index (Methods 2.4, p.103). |
| **C1** | Comparability: age + risk score / comorbidity index | ★ | Propensity score includes age and EuroSCORE II (Methods 2.5, p.103); 1:1 matching, doubly robust adjustment. |
| **C2** | Comparability: CAD complexity or LV function | ★ | LVEF included in the propensity model (Methods 2.5, p.103). |
| **O1** | Assessment of outcome | ★ | Record linkage with the Hospital Discharge Records database and Tax Registry for vital status (Methods 2.4, p.103); authors note outcomes were not VARC-defined (Discussion p.105). |
| **O2** | Follow-up long enough (≥30 days) | ★ | 30-day, 1-, 2- and 3-year outcomes (Tables 2–3, p.104). |
| **O3** | Adequacy of follow-up (≥90%) | ★ | 'Follow-up was complete in all patients' (Results 3.1, p.103). |
| **Total** | Selection 4 / Comparability 2 / Outcome 3 | **9/9** | **Quality: Good** |

## Baumbach 2019 (BMC Cardiovasc Disord 19:108)

| Item | Criterion | Score | Justification (page / section) |
|---|---|---|---|
| **S1** | Representativeness of exposed cohort (TAVR+PCI) | ★ | Prospective single-centre registry; all patients scheduled for AVR 2008–2016 'were consecutively enrolled' (Methods p.2–3). |

| Item | Criterion | Score | Justification (page / section) |
|---|---|---|---|
| **S2** | Selection of non-exposed cohort (SAVR+CABG) | ★ | SAVR+CABG from the same centre and period (Methods p.3); TAVR+OP/MIDCAB hybrid arm excluded from this review's counts. |
| **S3** | Ascertainment of exposure | ★ | Procedural records in the institutional registry (Methods p.3). |
| **S4** | Outcome not present at start | ★ | Outcomes are peri-/post-procedural by definition; prior MI within 90 d recorded as baseline (Definitions p.3). |
| **C1** | Comparability: age + risk score / comorbidity index | 0 | Early outcomes compared unadjusted across three groups (Table 3, p.5); adjusted Cox (age, EuroSCORE, LVEF, NYHA, pulmonary disease, PH, arrhythmia, CKD, AI, mitral/tricuspid disease) reported for 12-month mortality only (Results p.6) — scored 0 per protocol (same rule as Wendt). |
| **C2** | Comparability: CAD complexity or LV function | 0 | 0 — see C1. |
| **O1** | Assessment of outcome | 0 | Registry definitions; no VARC criteria, no adjudication; AKI 'stage II/III' with unspecified system, stroke row includes TIA (Table 3, p.5) — not reported. |
| **O2** | Follow-up long enough (≥30 days) | ★ | 30-day outcomes reported (Table 3, p.5). |
| **O3** | Adequacy of follow-up (≥90%) | 0 | Follow-up data available for 40.2% of TAVR+PCI and 53.2% of SAVR+CABG patients (Results 'Follow-up', p.6) — <90% and imbalanced. |
| **Total** | Selection 4 / Comparability 0 / Outcome 1 | **5/9** | **Quality: Poor** |

# Alperi 2021 (JACC Cardiovasc Interv 14:2490)

| Item | Criterion | Score | Justification (page / section) |
|---|---|---|---|
| **S1** | Representativeness of exposed cohort (TAVR+PCI) | ★ | Multicentre study of consecutive patients with severe AS and complex CAD (Abstract p.2490; Methods p.2491). |
| **S2** | Selection of non-exposed cohort (SAVR+CABG) | ★ | SAVR+CABG from the same centres and period, same inclusion criteria (Methods p.2491). |
| **S3** | Ascertainment of exposure | ★ | Dedicated database and in-hospital medical records (Methods p.2491). |
| **S4** | Outcome not present at start | ★ | Outcomes defined from the index procedure onward (Methods p.2491); MI defined per 4th universal definition. |
| **C1** | Comparability: age + risk score / comorbidity index | ★ | Propensity model includes age and STS score (Methods p.2492); 1:1 matching, 156 pairs. |
| **C2** | Comparability: CAD complexity or LV function | ★ | SYNTAX score, unprotected left main disease and LVEF in the propensity model (Methods p.2492). |
| **O1** | Assessment of outcome | ★ | Events defined according to VARC-2 (Methods p.2491); authors note no independent adjudication (Limitations p.2498). |
| **O2** | Follow-up long enough (≥30 days) | ★ | Median follow-up 3 years (Results p.2493). |
| **O3** | Adequacy of follow-up (≥90%) | ★ | 6 of 604 SAVR+CABG (1%) lost to follow-up; none in TAVR+PCI (Results p.2492). |
| **Total** | Selection 4 / Comparability 2 / Outcome 3 | **9/9** | **Quality: Good** |

# Patlolla 2022 (Ann Thorac Surg 114:659)

| Item | Criterion | Score | Justification (page / section) |
|---|---|---|---|
| **S1** | Representativeness of exposed cohort (TAVR+PCI) | ★ | All admissions in the HCUP National Inpatient Sample 2012–2017 meeting the code-based definition (Methods p.660). |
| **S2** | Selection of non-exposed cohort (SAVR+CABG) | ★ | SAVR+CABG admissions from the same database and years (Methods p.660). |
| **S3** | Ascertainment of exposure | ★ | ICD-9/ICD-10 procedure codes (Methods p.660; code list in supplement). |
| **S4** | Outcome not present at start | ★ | In-hospital outcomes coded as complications of the index admission (Methods p.660–661). |
| **C1** | Comparability: age + risk score / comorbidity index | 0 | Multivariable hierarchical logistic regression (age, sex, race, income, Charlson index, hospital factors, year) reported for in-hospital mortality only; all other outcomes compared unadjusted (Table 2, p.663, footnote 'unadjusted') — scored 0 per protocol. |
| **C2** | Comparability: CAD complexity or LV function | 0 | 0 — see C1; no CAD-complexity or LVEF variables available in NIS. |
| **O1** | Assessment of outcome | ★ | Outcomes identified by ICD diagnosis codes with a supplemental code list (Methods p.660); no code validation reported. |
| **O2** | Follow-up long enough (≥30 days) | 0 | In-hospital only — early outcomes truncated at discharge; no post-discharge follow-up (Limitations p.665). |
| **O3** | Adequacy of follow-up (≥90%) | ★ | Complete for the index admission by design (administrative data). |
| **Total** | Selection 4 / Comparability 0 / Outcome 2 | **6/9** | **Quality: Poor** |

# Elderia 2023 (Thorac Cardiovasc Surg 71:620)

| **Item** | **Criterion** | **Score** | **Justification (page / section)** |
|---|---|---|---|
| **S1** | Representativeness of exposed cohort (TAVR+PCI) | ★ | Monocentric observational analysis of consecutive patients with AS and CAD treated since 2017 (Methods p.621). |
| **S2** | Selection of non-exposed cohort (SAVR+CABG) | ★ | CABG+AVR patients from the same centre and period (Methods p.621). |
| **S3** | Ascertainment of exposure | ★ | Institutional records (Methods p.621). |
| **S4** | Outcome not present at start | ★ | Post-procedural outcomes; patients with prior valve surgery/recent MI excluded (Methods p.621). |
| **C1** | Comparability: age + risk score / comorbidity index | ★ | Propensity model includes age and EuroSCORE II (Methods p.622); 101 matched pairs. |
| **C2** | Comparability: CAD complexity or LV function | ★ | 3-vessel disease, left main involvement and LVEF <30% in the propensity model (Methods p.622). |
| **O1** | Assessment of outcome | ★ | VARC criteria applied to define endpoints (Methods p.621). |
| **O2** | Follow-up long enough (≥30 days) | ★ | 30-day outcomes (Table 6, p.625). |
| **O3** | Adequacy of follow-up (≥90%) | 0 | Completeness of 30-day follow-up not reported. |
| **Total** | Selection 4 / Comparability 2 / Outcome 2 | **8/9** | **Quality: Good** |

## Lérault 2023 (Arch Cardiovasc Dis 116:117)

| Item | Criterion | Score | Justification (page / section) |
|---|---|---|---|
| **S1** | Representativeness of exposed cohort (TAVR+PCI) | ★ | Consecutive patients with severe AS and concomitant CAD treated 2014–2020 at one centre (Methods 2.1, p.118). |
| **S2** | Selection of non-exposed cohort (SAVR+CABG) | ★ | SAVR+CABG from the same centre and period (Methods 2.1, p.118). |
| **S3** | Ascertainment of exposure | ★ | Medical records (Methods 2.3, p.118). |
| **S4** | Outcome not present at start | ★ | Post-procedural outcomes per VARC-3 (Methods 2.4, p.118). |
| **C1** | Comparability: age + risk score / comorbidity index | ★ | IPTW propensity score includes age and logistic EuroSCORE (Methods 2.5, p.119). Note: the IPTW-weighted comparison is the study's adjusted analysis; the HR estimated for this review from the raw KM curve is unadjusted, which is reflected in the HR dataset, not here. |
| **C2** | Comparability: CAD complexity or LV function | ★ | Number of diseased vessels, left main lesion and LVEF in the propensity score (Methods 2.5, p.119). |
| **O1** | Assessment of outcome | ★ | VARC-3 definitions (Methods 2.4, p.118). |
| **O2** | Follow-up long enough (≥30 days) | ★ | 30-day outcomes and median 27-month follow-up (Table 4, p.120; Results p.121). |
| **O3** | Adequacy of follow-up (≥90%) | 0 | Completeness of follow-up not reported (follow-up collected from records and by telephone, Methods p.118). |
| **Total** | Selection 4 / Comparability 2 / Outcome 2 | **8/9** | **Quality: Good** |

# Ullah 2023 (Catheter Cardiovasc Interv 102:946)

| Item | Criterion | Score | Justification (page / section) |
|---|---|---|---|
| **S1** | Representativeness of exposed cohort (TAVR+PCI) | ★ | All adult admissions in the Nationwide Readmissions Database 2015–2019 meeting ICD-10-CM criteria (Methods p.947). |
| **S2** | Selection of non-exposed cohort (SAVR+CABG) | ★ | SAVR+CABG from the same database and years, propensity-matched (Methods p.947–948). |
| **S3** | Ascertainment of exposure | ★ | ICD-10-CM procedure codes (Methods p.947; Supporting Table S1). |
| **S4** | Outcome not present at start | ★ | Index-admission complications coded after the procedure (Methods p.947). |
| **C1** | Comparability: age + risk score / comorbidity index | ★ | Propensity matching on age, sex, major comorbidities and Charlson index components, with further regression adjustment (Methods p.948; Table 3 note p.953). |
| **C2** | Comparability: CAD complexity or LV function | 0 | No CAD-complexity or LVEF variable in matching or adjustment (not available in NRD). |
| **O1** | Assessment of outcome | ★ | Outcome definitions by ICD-10 codes (Supporting Table S2, cited Methods p.947); no validation reported. |
| **O2** | Follow-up long enough (≥30 days) | 0 | Early outcomes are index-admission (truncated at discharge); 30-day data exist only for readmissions — scored 0 per protocol for administrative databases. |
| **O3** | Adequacy of follow-up (≥90%) | ★ | Complete for the index admission by design. |
| **Total** | Selection 4 / Comparability 1 / Outcome 2 | **7/9** | **Quality: Good** |

# Amat-Santos 2024 (JACC Cardiovasc Interv 17:2472)

| Item | Criterion | Score | Justification (page / section) |
|---|---|---|---|
| **S1** | Representativeness of exposed cohort (TAVR+PCI) | ★ | National multicentre registry (14 centres) enrolling consecutive patients 2018–2022 (Methods p.2473). |
| **S2** | Selection of non-exposed cohort (SAVR+CABG) | ★ | SAVR+CABG from the same centres and period (Methods p.2473). |
| **S3** | Ascertainment of exposure | ★ | Institutional records and dedicated database (Methods p.2474). |
| **S4** | Outcome not present at start | ★ | Outcomes post-procedural per VARC-3 (Methods p.2474). |
| **C1** | Comparability: age + risk score / comorbidity index | ★ | Propensity model includes age, EuroSCORE II and STS (Methods p.2474); 254 matched pairs. |
| **C2** | Comparability: CAD complexity or LV function | ★ | SYNTAX score and LVEF in the propensity model (Methods p.2474). |
| **O1** | Assessment of outcome | ★ | VARC-3 definitions (Methods p.2474); authors note no independent adjudication (Limitations p.2483). |
| **O2** | Follow-up long enough (≥30 days) | ★ | 30-day and 1-year outcomes (Supplemental Table 1). |
| **O3** | Adequacy of follow-up (≥90%) | ★ | Inclusion required completed 12-month follow-up (Methods p.2473) — complete by design; note this selects on follow-up. |
| **Total** | Selection 4 / Comparability 2 / Outcome 3 | **9/9** | **Quality: Good** |

# Jagadeesan 2025 (Ann Thorac Surg, 119:843)

| Item | Criterion | Score | Justification (page / section) |
|---|---|---|---|
| **S1** | Representativeness of exposed cohort (TAVR+PCI) | ★ | All Medicare beneficiaries ≥65 y with CAD undergoing PCI+TAVR 2018–2022 (Methods p.3). |
| **S2** | Selection of non-exposed cohort (SAVR+CABG) | ★ | CABG+SAVR beneficiaries from the same claims database and period (Methods p.3). |
| **S3** | Ascertainment of exposure | ★ | DRG and ICD-10-PCS procedure codes (Methods p.3). |
| **S4** | Outcome not present at start | ★ | 'Present on admission' modifier used to separate baseline conditions from complications (Methods p.3–4). |
| **C1** | Comparability: age + risk score / comorbidity index | ★ | IPW propensity weighting with 21 variables including age, Elixhauser score and frailty index, doubly robust regression (Methods p.4). |
| **C2** | Comparability: CAD complexity or LV function | ★ | Multivessel revascularization included as a covariate (Table 1, p.11) — accepted as a proxy for number of diseased vessels; no LVEF or SYNTAX available in claims. |
| **O1** | Assessment of outcome | ★ | Outcomes by ICD-10 codes with a supplemental code table (Methods p.3; Supplemental Table 2); no validation reported. |
| **O2** | Follow-up long enough (≥30 days) | 0 | Early outcomes are index-admission only (truncated at discharge); longitudinal claims follow-up applies to readmission outcomes — scored 0 per protocol. |
| **O3** | Adequacy of follow-up (≥90%) | ★ | Complete for index admission by design; longitudinal outcomes censored (Methods p.4). |
| **Total** | Selection 4 / Comparability 2 / Outcome 2 | **8/9** | **Quality: Good** |

# Taghiyev 2025 (J Card Surg 2025:3489054)

| Item | Criterion | Score | Justification (page / section) |
|---|---|---|---|
| **S1** | Representativeness of exposed cohort (TAVR+PCI) | ★ | 1232 consecutive patients at a tertiary centre 2010–2020 (Abstract p.1; Methods p.2). |
| **S2** | Selection of non-exposed cohort (SAVR+CABG) | ★ | SAVR+CABG from the same centre and period (Methods p.2). |
| **S3** | Ascertainment of exposure | ★ | Institutional database, prospectively collected (Methods p.2). |
| **S4** | Outcome not present at start | ★ | Patients with MI within 90 d, redo or concomitant procedures excluded (Methods p.3). |
| **C1** | Comparability: age + risk score / comorbidity index | ★ | Propensity model: age, STS and EuroSCORE (Methods p.3); 76 matched pairs. |
| **C2** | Comparability: CAD complexity or LV function | ★ | Ejection fraction and SYNTAX I score in the propensity model (Methods p.3). |
| **O1** | Assessment of outcome | ★ | Variables and outcomes per VARC-3 (Methods p.2); authors note events were self-adjudicated (Limitations p.9). |
| **O2** | Follow-up long enough (≥30 days) | ★ | 30-day outcomes and median 5.3-year follow-up (Results p.5). |
| **O3** | Adequacy of follow-up (≥90%) | ★ | 3.6% lost to follow-up (Results p.5). |
| **Total** | Selection 4 / Comparability 2 / Outcome 3 | **9/9** | **Quality: Good** |

# Stundl 2026 (Clin Res Cardiol 115:1188)

| Item | Criterion | Score | Justification (page / section) |
|---|---|---|---|
| **S1** | Representativeness of exposed cohort (TAVR+PCI) | ★ | Retrospective multicentre registry including all patients treated with TAVI+PCI or SAVR+CABG 2012–2020 meeting inclusion criteria (Methods p.1189–1190). |
| **S2** | Selection of non-exposed cohort (SAVR+CABG) | ★ | SAVR+CABG from the same centres and period (Methods p.1190). |
| **S3** | Ascertainment of exposure | ★ | Institutional records and registry database (Methods p.1190). |
| **S4** | Outcome not present at start | ★ | Outcomes recorded from the valve procedure onward per VARC-3 (Methods p.1190). |
| **C1** | Comparability: age + risk score / comorbidity index | ★ | Propensity model includes age and STS-PROM (Methods p.1191); 154 matched pairs. |
| **C2** | Comparability: CAD complexity or LV function | ★ | LVEF and main-stem disease in the propensity model (Methods p.1191). |
| **O1** | Assessment of outcome | ★ | VARC-3 criteria (Methods p.1190). |
| **O2** | Follow-up long enough (≥30 days) | ★ | 30-day to 3-year outcomes (Table 8, p.1196). |
| **O3** | Adequacy of follow-up (≥90%) | ★ | Follow-up adherence 98.1% at 1 year and 90.9% at 3 years (Discussion p.1196). |
| **Total** | Selection 4 / Comparability 2 / Outcome 3 | **9/9** | **Quality: Good** |

Table S5. Cochrane RoB 2 assessment of the included randomised trials

# 1. Søndergaard 2019 — SURTAVI revascularization substudy

| Signalling question | Response | Justification (page / section) |
|---|---|---|
| **Domain 1 — Bias arising from the randomisation process** | | |
| 1.1 Allocation sequence random? | No information | This paper states only that patients 'were stratified by center and the need for revascularization, and then were randomly assigned one-to-one to treatment with either TAVR or SAVR' (Methods, p.1298); the sequence-generation method is not described here (it is described in the parent SURTAVI publication, Reardon 2017, ref. 4). |
| 1.2 Sequence concealed until enrolment? | No information | Allocation concealment is not described in this paper (Methods, p.1298); enrolment decisions were made by a central SURTAVI screening committee before randomisation, which is reassuring but is not a statement about concealment. |
| 1.3 Baseline imbalances suggesting a problem? | Probably no | Table 1 (p.1299) compares the revascularization stratum with the non-revascularization stratum, not TAVR+PCI with SAVR+CABG; the only within-stratum comparison is coronary anatomy (Table 2, p.1299), which is balanced (SYNTAX 8.1 vs 8.5, P=0.45). No baseline table for the two randomised arms within the stratum is provided. |
| **Domain judgement** | **Some concerns** | Randomisation was stratified by need for revascularization, so the comparison of interest is a randomised comparison within a pre-defined stratum and the subgroup design does not by itself compromise Domain 1. However, sequence generation and concealment are not reported in this paper and no baseline comparison of the two randomised arms within the stratum is shown. |
| **Domain 2 — Bias due to deviations from intended interventions (effect of assignment)** | | |

| Signalling question | Response | Justification (page / section) |
|---|---|---|
| 2.1 Participants aware of assignment? | Yes | Surgical versus transcatheter treatment cannot be masked; no masking is described (Methods, p.1298). |
| 2.2 Carers / personnel aware? | Yes | As above; the revascularization strategy for TAVR patients 'was decided by the local Heart Team' (Methods, p.1298). |
| 2.3 Deviations due to trial context? | Probably no | 'Operators changed the assigned treatment for 109 patients (6.6%)... based on clinical judgment at the time of the scheduled procedure' (Results, p.1299); within the stratum 43/169 TAVR+PCI and 25/163 SAVR+CABG did not receive the planned revascularization (Figure 1, p.1298). These reflect usual clinical decision-making rather than the trial context. |
| 2.4 Deviations likely to affect outcome? | Probably no | Cancelling a planned PCI/CABG at a valve procedure is unlikely to change 30-day all-cause mortality materially; 30-day deaths were 7 vs 6 (Table 4, p.1300). |
| 2.5 Balanced between groups? | Probably no | Revascularization was cancelled in 25% of the TAVR+PCI arm vs 15% of the SAVR+CABG arm (Figure 1, p.1298). |
| 2.6 Appropriate analysis (ITT)? | Probably yes | Outcomes were compared 'by using the modified intention-to-treat population, which includes patients who had undergone randomization and an attempted procedure' (Methods, p.1298); the number randomised to the stratum but without an attempted procedure is not reported here. |
| 2.7 Substantial impact of not using ITT? | Not applicable | Analysis was by assigned group (mITT). |

| Signalling question | Response | Justification (page / section) |
|---|---|---|
| **Domain judgement** | **Some concerns** | Open-label with frequent, unbalanced non-receipt of the assigned revascularization, analysed by assigned group. The algorithm would permit 'Low' (deviations not attributable to trial context; ITT analysis), but the proportion of randomised patients in this stratum excluded before an attempted procedure is not reported, so Low is not assigned. |
| **Domain 3 — Bias due to missing outcome data** | | |
| 3.1 Data available for all or nearly all? | No information | Completeness of 30-day follow-up in the revascularization stratum is not stated; Table 4 (p.1300) reports 30-day events for 169 and 163 patients without a statement on missing vital status. |
| 3.2 Evidence result not biased by missingness? | No information | No sensitivity analysis for missing data is reported. |
| 3.3 Missingness could depend on true value? | Probably no | Vital status at 30 days in a monitored device trial with an independent CEC (p.1298) is unlikely to be missing differentially. |
| 3.4 Likely depends on true value? | Probably no | As above. |
| **Domain judgement** | **Some concerns** | Missing data are unlikely for 30-day mortality in SURTAVI, but the paper does not report completeness of follow-up for this stratum. |
| **Domain 4 — Bias in measurement of the outcome** | | |
| 4.1 Inappropriate method? | No | All-cause mortality is objective; 'events were adjudicated by an independent clinical events committee using VARC-2 criteria' (Methods, p.1298). |

| Signalling question | Response | Justification (page / section) |
|---|---|---|
| 4.2 Differed between groups? | No | Same adjudication process for both arms (p.1298). |
| 4.3 Assessors aware of assignment? | Probably yes | Masking of the CEC is not stated; with surgery vs TAVR, awareness is likely. |
| 4.4 Assessment could be influenced? | No | Death is not open to assessor influence. |
| 4.5 Likely influenced? | Not applicable | — |
| **Domain judgement** | **Low** | Objective outcome with independent adjudication. |
| **Domain 5 — Bias in selection of the reported result** | | |
| 5.1 Analysed per prespecified plan? | No information | The authors call this 'a prespecified subgroup analysis of the SURTAVI trial' (Discussion, p.1301) and cite NCT01586910 (Abstract, p.1296), but no protocol or SAP for the substudy is cited and the registered outcomes belong to the parent trial (TAVR vs SAVR); the primary endpoint of the substudy is death or disabling stroke at 2 years, with 30-day all-cause mortality a component reported in Table 4. |
| 5.2 Selected from multiple measurements? | Probably no | All-cause mortality is reported at both time points assessed (30 days, Table 4 p.1300; 2 years, Table 5 p.1301). |

| Signalling question | Response | Justification (page / section) |
| --- | --- | --- |
| 5.3 Selected from multiple analyses? | Probably no | Both the mITT (Table 4) and the as-treated (Table 7, p.1302) analyses are reported, with mITT prespecified as primary (Methods, p.1298). |
| **Domain judgement** | **Some concerns** | Prespecification is asserted but not verifiable against a substudy protocol/SAP; the comparison of interest is a secondary component within a subgroup of a trial registered for a different primary comparison. |

# 2. Kedhi 2024 — TCW

| Signalling question | Response | Justification (page / section) |
| --- | --- | --- |
| **Domain 1 — Bias arising from the randomisation process** | | |
| 1.1 Allocation sequence random? | Yes | '...according to a computer-generated sequence with random permuted blocks sizes stratified by site' (Randomisation and masking, p.2595). |
| 1.2 Sequence concealed until enrolment? | Yes | Assignment was made 'using a dedicated website as part of the electronic case report form' (p.2595), i.e. central web-based allocation. |
| 1.3 Baseline imbalances suggesting a problem? | No | 'Baseline characteristics were well-balanced between the groups (table 1)' (Results, p.2597; Table 1, p.2596). |

| Signalling question | Response | Justification (page / section) |
|---|---|---|
| **Domain judgement** | **Low** | Adequate generation and concealment; balanced baseline. |
| **Domain 2 — Bias due to deviations from intended interventions (effect of assignment)** | | |
| 2.1 Participants aware of assignment? | Yes | 'Patients, investigators, and members of the clinical event adjudication committee were not masked to group assignment' (p.2595). |
| 2.2 Carers / personnel aware? | Yes | As above (open-label trial, title and p.2595). |
| 2.3 Deviations due to trial context? | Probably yes | Crossover from surgery to FFR-guided PCI+TAVI (7 of 81, plus 1 PCI-only and 1 TAVI-only; Figure 1, p.2595) was 'partly influenced by... the patient preference for a less invasive procedure' after allocation (Discussion, p.2601); 2 of 91 TAVI patients deviated. |
| 2.4 Deviations likely to affect outcome? | Probably no | With 0 vs 1 deaths at 30 days (Table 3, p.2600) the crossovers cannot have changed the early-mortality estimate materially; any effect would bias toward the null (surgical-arm patients receiving TAVI). |
| 2.5 Balanced between groups? | No | 9/81 deviations in the surgical arm vs 2/91 in the percutaneous arm (Figure 1, p.2595). |
| 2.6 Appropriate analysis (ITT)? | Yes | 'The primary and safety analyses were done per an intention-to-treat principle' (Abstract, p.2593; Statistical analysis, p.2597), with per-protocol and as-treated analyses as supportive evidence. |

| Signalling question | Response | Justification (page / section) |
|---|---|---|
| 2.7 Substantial impact of not using ITT? | Not applicable | ITT used. |
| **Domain judgement** | **Some concerns** | Open-label trial with unbalanced, partly preference-driven crossover (11% vs 2%). Analysis by ITT and the deviations bias toward the null for this outcome, so the algorithm would allow 'Low'; 'Some concerns' is retained because the supportive per-protocol/as-treated results are in the appendix only and the impact on a 0 vs 1 event count cannot be verified from the paper. |
| **Domain 3 — Bias due to missing outcome data** | | |
| 3.1 Data available for all or nearly all? | Probably no | 'Of 81 patients in the SAVR plus CABG group, four died before the procedure and were not included in the intention-to-treat analysis' (Results, p.2597; Figure 1 footnote, p.2595: 'as mandated per protocol'); 'No patients were lost to follow-up' (p.2597). Four post-randomisation exclusions (4.9%) confined to one arm, and the exclusion is itself the outcome. |
| 3.2 Evidence result not biased by missingness? | Probably yes | 'The safety analysis was done per intention to treat, which included all events starting from enrolment' (p.2597) and the authors state the ITT findings 'were further corroborated by the findings of the per-protocol analysis, safety analysis, and as-treated analysis' (Discussion, p.2599); the numbers are in the appendix (not in the folder). Including the four deaths would strengthen, not weaken, the difference favouring TAVI. |
| 3.3 Missingness could depend on true value? | Yes | The excluded patients were excluded because they died. |

| Signalling question | Response | Justification (page / section) |
|---|---|---|
| 3.4 Likely depends on true value? | Yes | As above. |
| **Domain judgement** | **Some concerns** | Exclusion of four pre-procedure deaths from the surgical arm is outcome-dependent exclusion in one arm. The direction of bias is against the reported finding (it removes surgical-arm deaths) and a corroborating safety analysis is described, so the algorithm would give 'Low' via 3.2; 'Some concerns' is assigned because the corroborating numbers are not reported in the main paper. |
| **Domain 4 — Bias in measurement of the outcome** | | |
| 4.1 Inappropriate method? | No | All-cause mortality; 'Adverse events were adjudicated by an independent clinical event committee' (p.2595). |
| 4.2 Differed between groups? | No | Same process for both arms. |
| 4.3 Assessors aware of assignment? | Yes | '...members of the clinical event adjudication committee were not masked to group assignment' (p.2595). |
| 4.4 Assessment could be influenced? | No | Death is not open to assessor influence; the open-label design does not matter for this outcome. |
| 4.5 Likely influenced? | Not applicable | — |

| Signalling question | Response | Justification (page / section) |
|---|---|---|
| **Domain judgement** | **Low** | Objective outcome. |
| **Domain 5 — Bias in selection of the reported result** | | |
| 5.1 Analysed per prespecified plan? | Probably no | Registered (NCT03424941, p.2593). However, 'enrolment was stopped as a safety data analysis (done in 33% of patients and repeated at 50% of enrolment vs 66% in the protocol) showed an important difference between the two groups (p<0·0001) for the primary endpoint and all-cause mortality' (Statistical analysis, p.2596): the interim look that stopped the trial was performed earlier than the protocol specified, and mortality was one of the triggers. |
| 5.2 Selected from multiple measurements? | Probably no | All-cause mortality reported at both scheduled visits (30 days and 1 year; Table 3, p.2600). |
| 5.3 Selected from multiple analyses? | Probably no | ITT prespecified as primary, with per-protocol, as-treated and safety analyses all reported (p.2597, p.2599). |
| **Domain judgement** | **Some concerns** | Early termination at ~50% of planned enrolment (172 of 320) after an interim analysis performed off-schedule, with all-cause mortality among the stopping triggers; trials stopped early for benefit tend to overestimate the effect. |

## 3. Summary

| **Domain** | **Søndergaard 2019 (SURTAVI substudy)** | **Kedhi 2024 (TCW)** |
|---|---|---|
| Domain 1 — Randomisation process | Some concerns | Low |
| Domain 2 — Deviations from intended interventions | Some concerns | Some concerns |
| Domain 3 — Missing outcome data | Some concerns | Some concerns |
| Domain 4 — Measurement of the outcome | Low | Low |
| Domain 5 — Selection of the reported result | Some concerns | Some concerns |
| **Overall** | Some concerns | Some concerns |

**Table S6.** GRADE summary of findings: TAVR+PCI versus SAVR+CABG.

| Outcome | Studies (participants) | Certainty assessment | | | | | Relative effect (95% CI) | Assumed risk with SAVR+CABG; difference with TAVR+PCI, per 1000 (95% CI) g | Certainty |
|---|---|---|---|---|---|---|---|---|---|
| | | Risk of bias | Inconsistency | Indirectness | Imprecision | Other considerations | | | |
| Early all-cause mortality (in-hospital or 30-day) | 2 RCT, 11 observational (154,580) | Not serious a | Not serious b | Not serious | Not serious | None | OR 0.60 (0.44–0.84) | 52 per 1000 20 fewer (28 to 8 fewer) | ⊕⊕○○ Low |
| One-year all-cause mortality | 1 RCT, 3 observational (1,456) | Not serious a | Not serious | Not serious | Serious c | None | OR 0.80 (0.58–1.10) | 129 per 1000 23 fewer (50 fewer to 11 more) | ⊕○○○ Very low |
| All-cause mortality over follow-up (12–72 months) | 9 observational (40,634) | Not serious a | Not serious | Not serious d | Not serious | One study carries 76% of weight; result unchanged without it | HR 1.38 (1.28–1.49) | Not estimated (variable follow-up) | ⊕⊕○○ Low |
| Permanent pacemaker | 2 RCT, 11 observational (154,580) | Not serious a | Not serious | Not serious | Not serious | Large effect, consistent across designs (+1) e | OR 2.18 (1.93–2.46) | 52 per 1000 54 more (43 to 66 more) | ⊕⊕⊕○ Moderate |
| Major vascular complications | 2 RCT, 8 observational (142,400) | Not serious a | Serious f | Not serious | Not serious | Large effect (+1) e | OR 3.38 (1.65–6.89) | 10 per 1000 23 more (6 to 55 more) | ⊕⊕○○ Low |
| Stroke | 2 RCT, 11 observational (154,580) | Not serious a | Not serious | Not serious h | Not serious i | None | OR 0.88 (0.78–1.00) | 29 per 1000 3 fewer (6 fewer to 0) | ⊕⊕○○ Low |

| Outcome | Studies (participants) | Certainty assessment | | | | | Relative effect (95% CI) | Assumed risk with SAVR+CABG; difference with TAVR+PCI, per 1000 (95% CI) g | Certainty |
|---|---|---|---|---|---|---|---|---|---|
| | | Risk of bias | Inconsistency | Indirectness | Imprecision | Other considerations | | | |
| Major or life-threatening bleeding | 2 RCT, 6 observational<br><br>(51,212) | Not serious a | Very serious j | Serious k | Serious c | None | OR 0.53 (0.28–1.02) | 122 per 1000<br><br>53 fewer (85 fewer to 2 more) | ⊕◯◯◯<br><br>Very low |
| Acute kidney injury | 1 RCT, 7 observational<br><br>(142,454) | Not serious a | Serious f | Not serious l | Not serious | Large effect (+1) e | OR 0.34 (0.20–0.57) | 140 per 1000<br><br>88 fewer (108 to 55 fewer) | ⊕⊕◯◯<br><br>Low |
| Myocardial infarction | 2 RCT, 5 observational<br><br>(2,516) | Not serious a | Not serious | Serious m | Very serious n | None | OR 0.65 (0.31–1.35) | 13 per 1000<br><br>5 fewer (9 fewer to 4 more) | ⊕◯◯◯<br><br>Very low |
| Repeat revascularization (exploratory) | 1 RCT, 4 observational<br><br>(39,122) | Not serious a | Serious o | Serious p | Not serious | None | OR 3.04 (1.28–7.26) | Not estimated (variable follow-up) | ⊕◯◯◯<br><br>Very low |

TAVR+PCI compared with SAVR+CABG (reference) in patients with severe aortic stenosis and coronary artery disease. OR or HR < 1 favours TAVR+PCI. Evidence from non-randomized studies starts at low certainty; the two randomized sources contributed a minority of the weight for every outcome (1–22%) and did not change the starting level.

a. Confounding by indication is inherent to the observational evidence and is reflected in the low starting certainty; no additional downgrade. [Align with the ROBINS-I/RoB 2 table.]

b. $I^2 = 79\%$, arising entirely from discordant administrative databases; excluding them gave the same estimate with $I^2 = 0\%$ (OR 0.63, 0.44–0.89). Not downgraded.

c. The confidence interval includes both appreciable benefit and no effect (or harm).

d. Follow-up ranged from 12 to 72 months and hazards were not proportional; four hazard ratios were estimated from Kaplan–Meier data. Judged not serious because results were unchanged in studies with ≥24 months of follow-up and in reported-only estimates including the Medicare cohort.

e. Upgraded one level: OR > 2 (or < 0.5), same direction in randomized, cohort and administrative strata, and unchanged after excluding administrative databases.

f. $I^2 \geq 80\%$ in the primary analysis and 43–66% after excluding administrative databases; direction consistent, magnitude variable.

g. The assumed risk is the median SAVR+CABG event rate across contributing studies with available event counts; where counts were not tabulated they were derived from the reported odds ratio, confidence interval and group sizes. Adjusted-only estimates (Jagadeesan et al.) did not contribute to the assumed risk.

h. Stroke was ICD-coded in the three databases carrying 92% of the weight; results were similar in clinically adjudicated studies (OR 0.72, 0.44–1.20).

i. The confidence interval reaches no effect but excludes appreciable harm and does not include a relative reduction larger than 25%.

j. $I^2 = 95\%$; estimates ranged from OR 0.17 to 1.95, and the two randomized sources and the two administrative databases each pointed in opposite directions.

k. Definitions differed (VARC-2, VARC-3, transfusion-based, ICD-coded) and treat surgical transfusion differently.

l. Most studies counted acute kidney injury of any stage; the effect on stage II–III injury is uncertain (one study, OR 0.94).

m. Periprocedural myocardial infarction is defined by different biomarker thresholds after PCI and after CABG.

n. 33 events in total; the confidence interval spans a 69% reduction to a 35% increase.

o. $I^2 = 56\%$; the estimate was significant only with the Medicare cohort included (without it OR 1.91, 0.82–4.43).

p. Follow-up ranged from 1 to more than 5 years; odds ratios do not account for time at risk or the competing risk of death.

CI, confidence interval; HR, hazard ratio; OR, odds ratio; RCT, randomized controlled trial; VARC, Valve Academic Research Consortium.

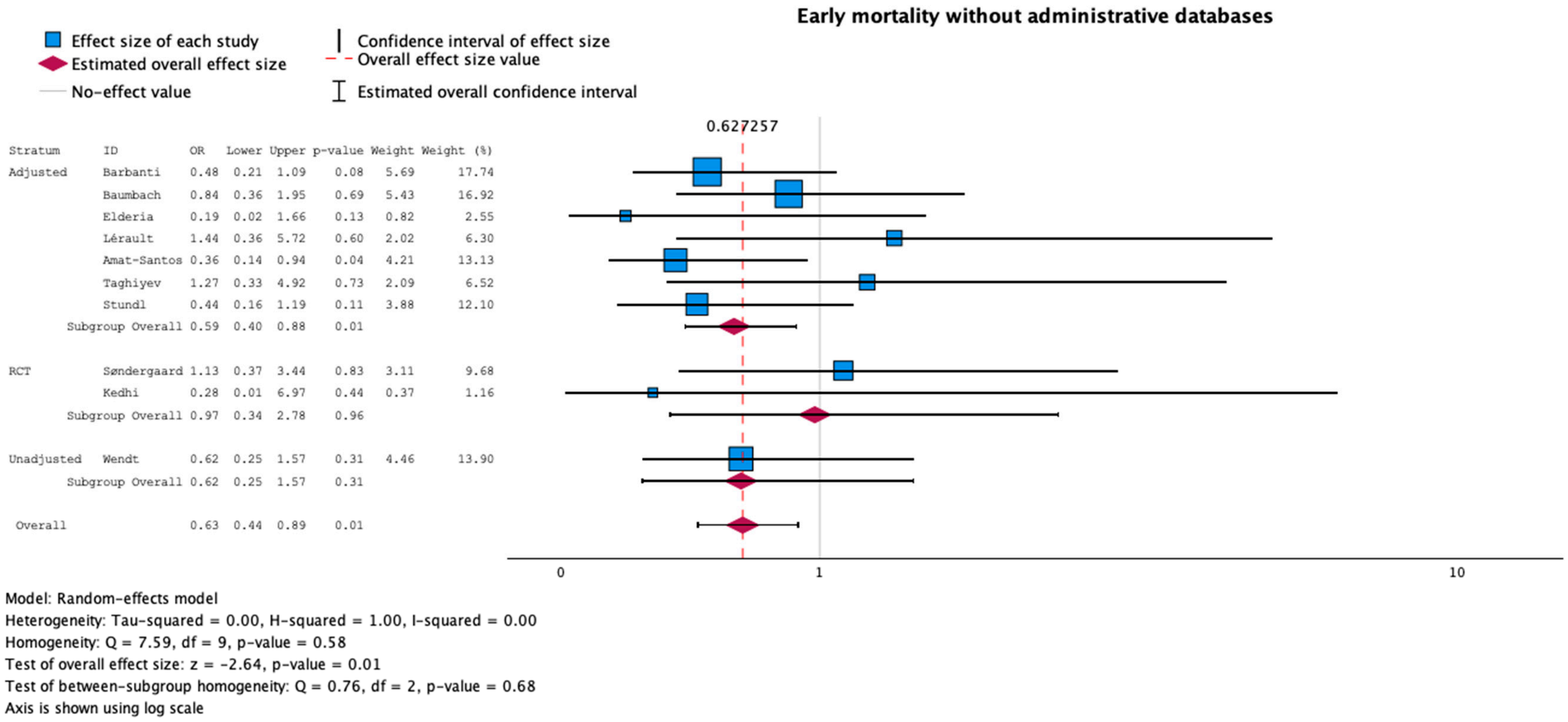


**Figure S1.** Early all-cause mortality after TAVR+PCI versus SAVR+CABG, excluding administrative databases, by study design. Random-effects model (REML); SAVR+CABG is the reference, and OR < 1 favours TAVR+PCI.

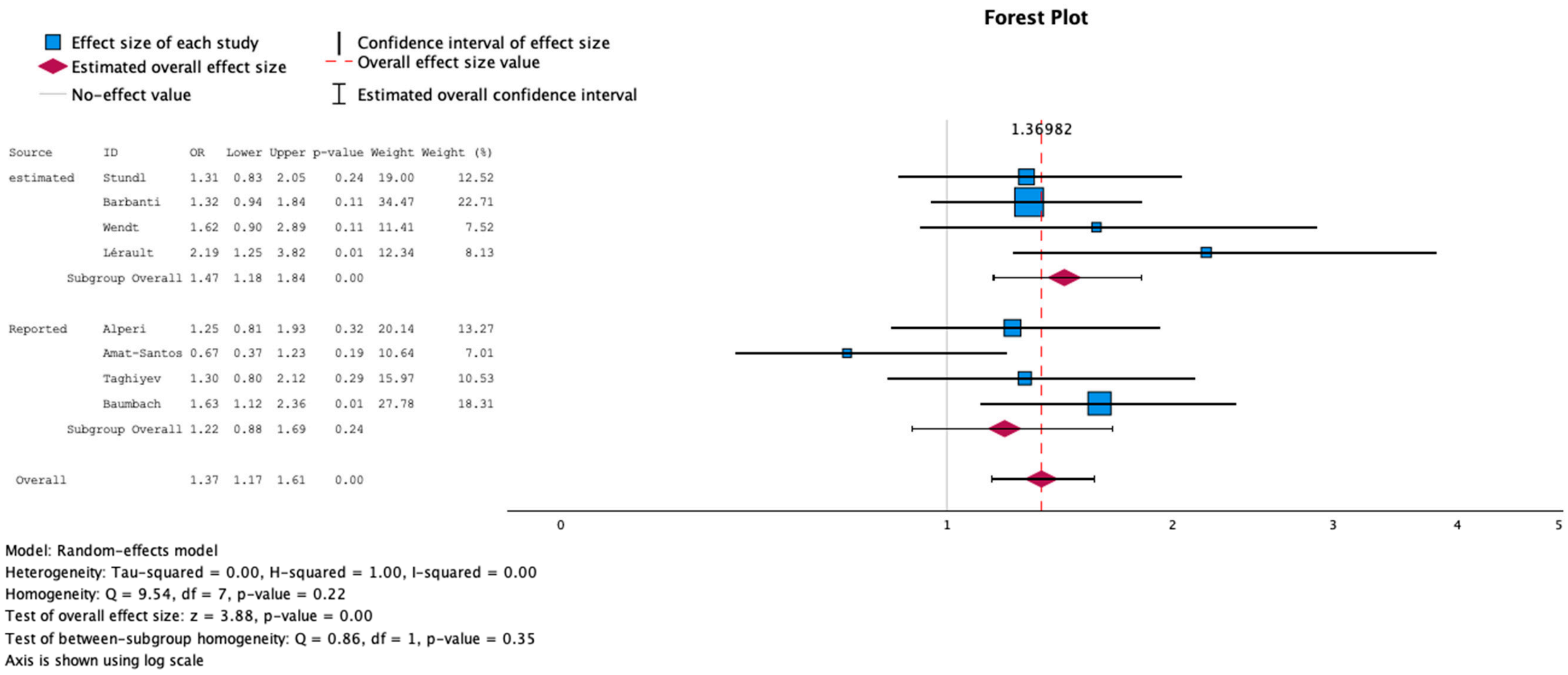


**Figure S2.** All-cause mortality over follow-up after TAVR+PCI versus SAVR+CABG, excluding the Medicare cohort (Jagadeesan et al.), by source of the hazard ratio (reported by the original authors or estimated from Kaplan–Meier data). Random-effects model (REML); SAVR+CABG is the reference, and HR > 1 favours SAVR+CABG. Diamonds show pooled estimates for each source and overall.

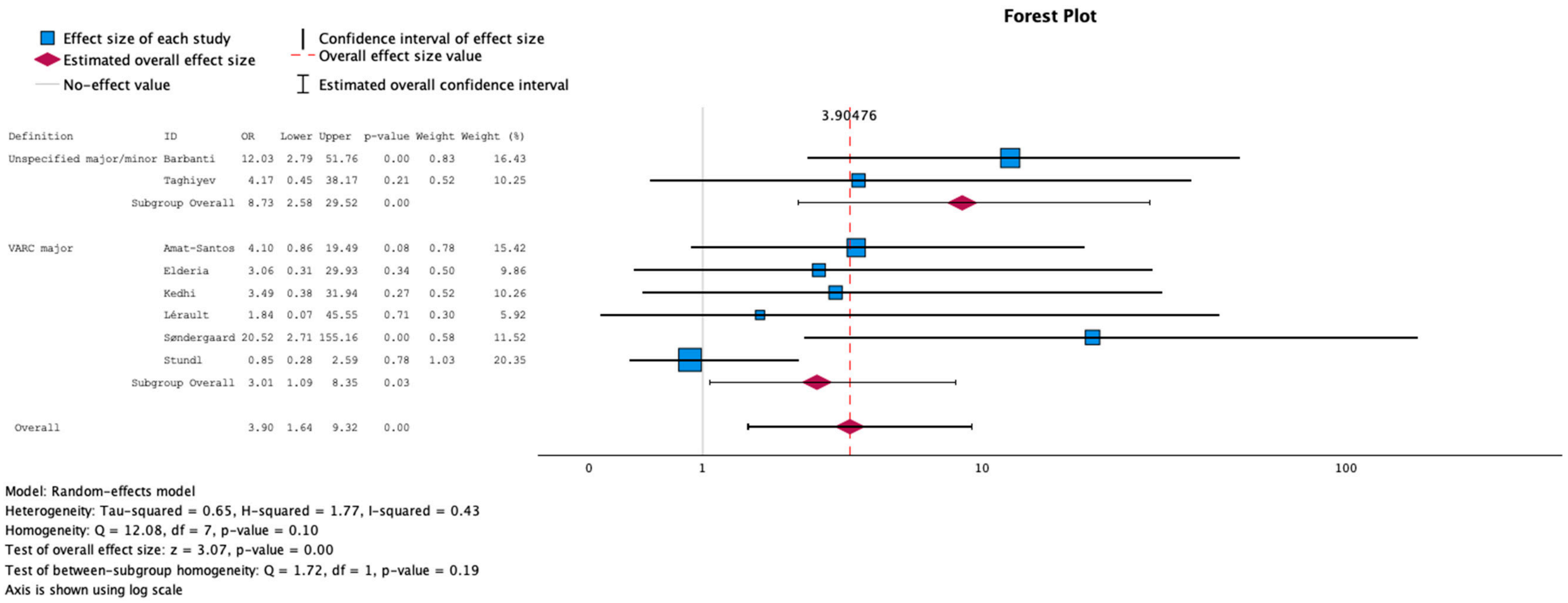


**Figure S3.** Major vascular complications after TAVR+PCI versus SAVR+CABG, excluding administrative databases, by outcome definition. Random-effects model (REML); SAVR+CABG is the reference, and OR > 1 favours SAVR+CABG. Diamonds show pooled estimates for each definition and overall.

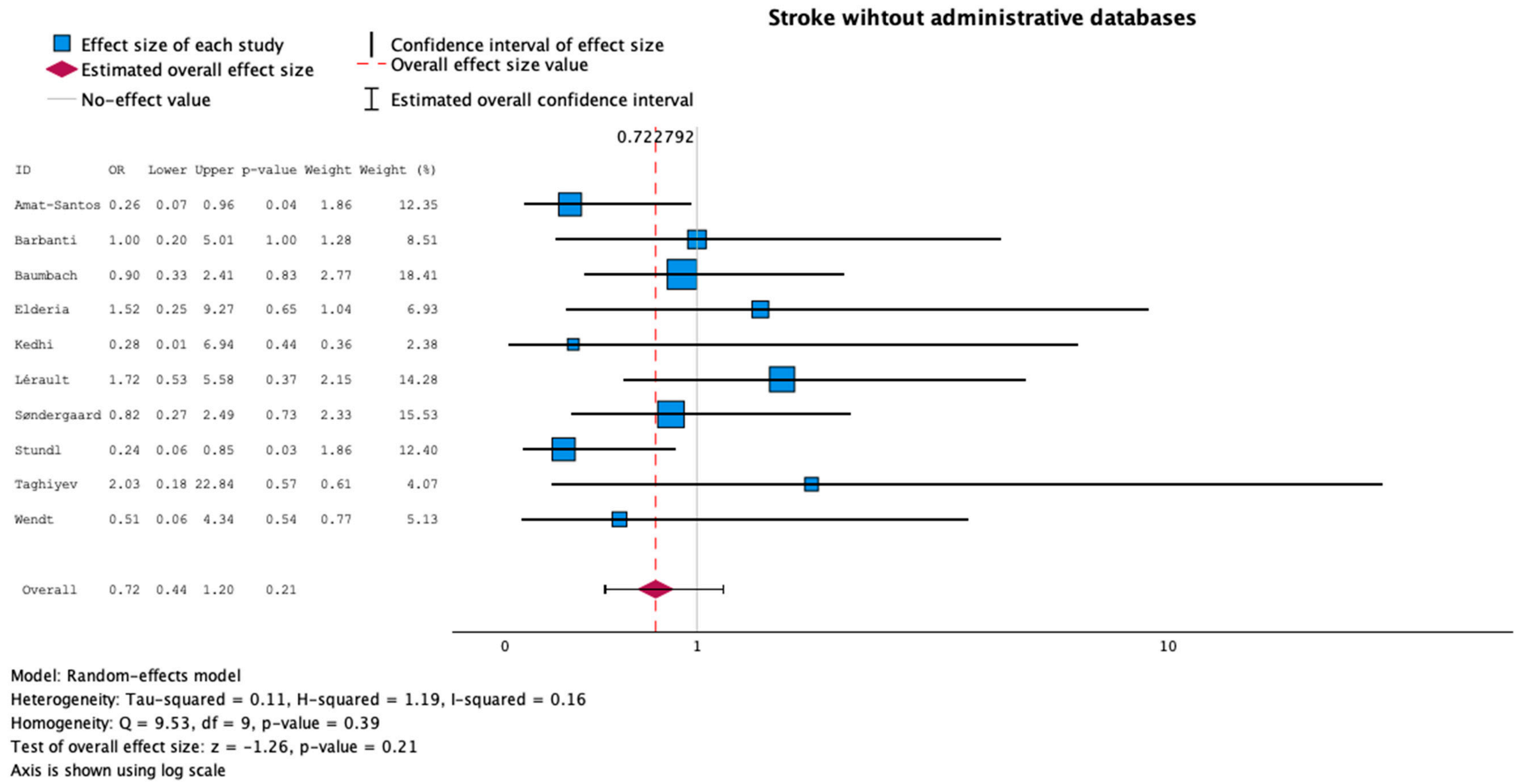


**Figure S4.** Early stroke after TAVR+PCI versus SAVR+CABG, excluding administrative databases. Random-effects model (REML); SAVR+CABG is the reference, and OR < 1 favours TAVR+PCI.

| Definition | ID | LogOR | Std. Error | Lower | Upper | p-value | Weight | Weight (%) |
|---|---|---|---|---|---|---|---|---|
| Registry (>4 U RBC) | Barbanti | -1.64 | 0.40 | -2.42 | -0.85 | 0.00 | 1.29 | 16.94 |
| | Subgroup Overall | -1.64 | 0.40 | -2.42 | -0.85 | 0.00 | | |
| VARC major/LT | Kedhi | -1.78 | 0.66 | -3.08 | -0.49 | 0.01 | 0.95 | 12.52 |
| | Søndergaard | 0.35 | 0.34 | -0.31 | 1.01 | 0.30 | 1.37 | 18.05 |
| | Subgroup Overall | -0.64 | 1.07 | -2.73 | 1.45 | 0.55 | | |
| VARC-3 life-threatening | Lérault | -1.03 | 0.50 | -2.02 | -0.05 | 0.04 | 1.15 | 15.16 |
| | Subgroup Overall | -1.03 | 0.50 | -2.02 | -0.05 | 0.04 | | |
| VARC-3 major | Amat-Santos | -0.29 | 0.29 | -0.85 | 0.28 | 0.32 | 1.43 | 18.86 |
| | Stundl | -1.59 | 0.31 | -2.21 | -0.98 | 0.00 | 1.40 | 18.47 |
| | Subgroup Overall | -0.94 | 0.65 | -2.22 | 0.35 | 0.15 | | |
| Overall | | -0.94 | 0.36 | -1.65 | -0.23 | 0.01 | | |

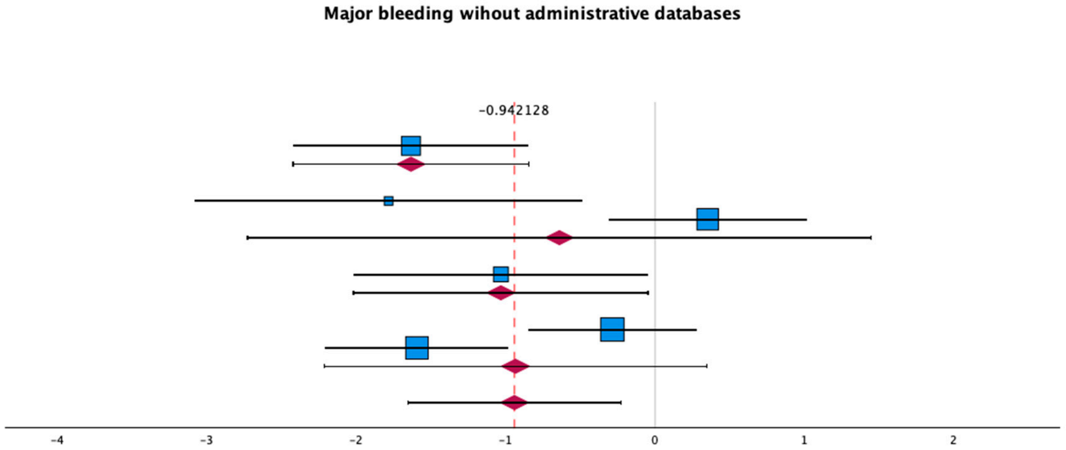


Model: Random-effects model
Heterogeneity: Tau-squared = 0.62, H-squared = 5.26, I-squared = 0.81
Homogeneity: Q = 27.99, df = 5, p-value = 0.00
Test of overall effect size: z = -2.60, p-value = 0.01
Test of between-subgroup homogeneity: Q = 1.65, df = 3, p-value = 0.65

**Figure S5.** Early major or life-threatening bleeding after TAVR+PCI versus SAVR+CABG, excluding administrative databases, by outcome definition. Random-effects model (REML) on the log odds ratio scale; SAVR+CABG is the reference, and values < 0 (OR < 1) favour TAVR+PCI. Diamonds show pooled estimates for each definition and overall.
